# TrkA promotes MDM2-mediated AGPS ubiquitination and degradation to trigger prostate cancer progression

**DOI:** 10.1186/s13046-023-02920-w

**Published:** 2024-01-11

**Authors:** Yu Zhang, Zhenlin Huang, Keqiang Li, Guoqing Xie, Yuankang Feng, Zihao Wang, Ningyang Li, Ruoyang Liu, Yinghui Ding, Jun Wang, Jinjian Yang, Zhankui Jia

**Affiliations:** 1 Department of Urology, the First Affiliated Hospital of Zhengzhou University, Zhengzhou, 450052 China; 2 Biochemistry and Molecular Biology, Mayo Clinic, Rochester, MN 55905 USA; 3 Department of Otorhinolaryngology, the First Affiliated Hospital of Zhengzhou University, Zhengzhou, 450052 China; 4 Academy of Medical Science, Zhengzhou University, Zhengzhou, 450052 China

**Keywords:** Prostate cancer, Ferroptosis, AGPS, MDM2, TrkA

## Abstract

**Background:**

As a novel necrosis manner, ferroptosis has been increasingly reported to play a role in tumor progression and treatment, however, the specific mechanisms underlying its development in prostate cancer remain unclear. Growing evidence showed that peroxisome plays a key role in ferroptosis. Herein, we identified a novel mechanism for the involvement of ferroptosis in prostate cancer progression, which may provide a new strategy for clinical treatment of prostate cancer.

**Methods:**

Label-Free Mass spectrometry was used to screen and identify candidate proteins after ferroptosis inducer-ML210 treatment. Immunohistochemistry was undertaken to explore the protein expression of AGPS in prostate cancer tissues compared with normal tissues. Co-immunoprecipitation and GST pull-down were used to identify the directly binding of AGPS to MDM2 in vivo and in vitro. CCK8 assay and colony formation assay were used to illustrate the key role of AGPS in the progression of prostate cancer in vitro. The xenograft model was established to verify the key role of AGPS in the progression of prostate cancer in vivo.

**Results:**

AGPS protein expression was downregulated in prostate cancer tissues compared with normal tissues from the first affiliated hospital of Zhengzhou University dataset. Lower expression was correlated with poorer overall survival of patients compared to those with high expression of AGPS. In addition, AGPS can promote ferroptosis by modulating the function of peroxisome-resulting in the lower survival of prostate cancer cells. Furthermore, it was shown that AGPS can be ubiquitinated and degraded by the E3 ligase-MDM2 through the proteasomal pathway. Meanwhile, kinase TrkA can promote the combination of AGPS and MDM2 by phosphorylating AGPS at Y451 site. It was verified that kinase TrkA inhibitor—Larotrectinib can increase the susceptibility of prostate cancer cells to ferroptosis, which leads to the inhibition of prostate cancer proliferation to a great extent in vitro and in vivo.

**Conclusion:**

Based on these findings, we proposed the combination of ferroptosis inducer and TrkA inhibitor to synergistically exert anti-tumor effects, which may provide a new strategy for the clinical treatment of prostate cancer.

**Supplementary Information:**

The online version contains supplementary material available at 10.1186/s13046-023-02920-w.

## Introduction

Prostate cancer (PCa) is the most common malignancy in men, which has the second highest fatality rate among male tumors and accounts for 20% of annually diagnosed cancer [1]. In recent years, with aging and changes in dietary habits, the incidence of PCa has increased rapidly [2–4]. Androgen deprivation therapy (ADT) has become the standard of care for patients with advanced PCa [5, 6]. However, PCa cells gradually become insensitive to ADT treatment and eventually develop into castration-resistant PCa (CRPC) [7], and the current radiotherapy or drug treatment of CRPC is ineffective and non-specific [8]. Therefore, the exploration of new potential therapeutic targets for PCa treatment is particularly important.

Ferroptosis is a unique iron-dependent and non-apoptotic form of necrosis due to lipid peroxidation. Several studies have reported that ferroptosis is involved in various degenerative diseases and tumors [9, 10]. Recent studies have displayed that peroxisome plays an important role in the occurrence of ferroptosis via lipid peroxidation by promoting the production of PUFAs and inhibiting the reduction of PUFAs [11, 12]. The discovery of the ferroptosis peroxisome-dependent pathway gradually revealed the importance of ether phospholipids (ePLs) in ferroptosis [13, 14].

Mouse double minute 2 (MDM2) has been shown to act as an oncogene in a variety of tumors, including prostate cancer. Not only is it a negative regulator of p53, but it has also been associated with cancer’s poor prognosis, high recurrence probability, and resistance to treatment. Targeted MDM2 therapy is believed to increase the sensitivity of prostate carcinoma to androgen deprivation therapy (ADT) in a p53-dependent or independent manner, so targeted MDM2 therapy is increasingly a promising treatment for clinical tumors [15].

Alkylglycerone phosphate synthase (AGPS) is a key enzyme in the synthesis of ether lipids to promote peroxisome formation and increase the sensitivity of tumor cells to ferroptosis [16]. Daolin Tang [17] have previously summarized on the significant involvement of peroxisomes in the process of ferroptosis. Specifically, peroxisomes play a crucial role in ferroptosis by synthesizing polyunsaturated ether phospholipids (PUFA-ePLs), which serve as substrates for lipid peroxidation. This lipid peroxidation process ultimately leads to the induction of ferroptosis. Additionally, AGPS has been identified as a key contributor to lipid peroxidation by facilitating the formation and maturation of peroxisomes. This, finally, promotes the generation of the necessary substrate for lipid peroxidation- PUFA-ePLs. Consequently, the relationship between AGPS and the development of PCa may be related to ferroptosis.

In this study, we revealed that the E3 ligase MDM2 can promote the ubiquitination and degradation of AGPS in a p53-independent manner. Meanwhile, the degradation of AGPS was promoted by tyrosine kinase receptor A (TrkA), which could modulate the progression of PCa by modifying the phosphorylation of AGPS to enhance the function of MDM2 on AGPS ubiquitination and degradation. The TrkA inhibitor could protect AGPS and promote ferroptosis in prostate cancer and exert a better anti-cancer function when combined with a ferroptosis inducer.

## Materials and methods

### Cell lines, cell culture, and transfection

DU145, 22Rv1, C4-2, and 293 T cell lines were purchased from Cell Bank of Chinese Academy of Sciences (Shanghai, China). 22Rv1 and C4-2 cells were cultured in RPMI-1640 medium, 293 T were cultured in DMEM medium, and DU145 were cultured in MEM medium, all mediums supplemented with 10% FBS. The cells were cultured for use in the logarithmic growth phase. Lipofectamine 2000 (Thermo Fisher Scientific) was used to perform transfection following the manufacturer’s protocol.

### Lable-free mass spectrometry

500 μL of 8 M urea was added to the DU145 cell samples and the protease inhibitor was added at 10% of the lysate. After centrifuging at 14,100 × g for 20 min, the supernatant was collected. Adding 200 mM dithiothreitol (DTT) solution and incubate at 37 °C for 1 h. The sample was diluted 4 times by adding 25 mM ammonium bicarbonate (ABC) buffer. Then adding trypsin (trypsin: protein = 1:50) and incubate at 37℃ overnight. The next day, adding 50μL 0.1% FA was to terminate the digestion. Take 100 μl 100% ACN to wash the C18 column, and centrifuge at 1200 rpm for 3 min. Wash the column once with 100 μl of 0.1% FA and centrifuge at 1200 rpm for 3 min. Replace the EP tube, add the sample, and centrifuge at 1200 rpm for 3 min. Wash the column twice with 100 μl of 0.1% FA and centrifuge at 1200 rpm for 3 min. Wash once with 100 μl of pH 10 water. Replace the EP tube and elute with 70% ACN. The eluents of each sample were combined and lyophilized. Store at -80 °C until loading. Nanoflow LC–MS/MS analysis of tryptic peptides was conducted on the Thermo Scientific Orbitrap Exploris™480 platform coupled to an EASY nLC 1200 ultra-high-pressure system (Thermo Fisher Scientific) via a nano-electrospray ion source. 500 ng of peptides were loaded on a 25 cm column (150 μm inner diameter, packed using ReproSil-Pur C18-AQ 1.9- µm silica beads; Beijing Qinglian Biotech Co., Ltd, Beijing, China). Peptides were eluted over a 120 min gradient at a flow rate of 600 nl/min, using 80% Acetonitrile, 0.1% Formic acid (Buffer B) going from 8 to 12% over 10 min, to 30% over 79 min, then to 40% over 16 min and 95% over 1 min, and holding it at 95% for 4 min. MS2 spectra were searched against the UniProtKB human proteome database human reference protein sequences (20,373 target sequences downloaded on 17 March 2022). The Sequest HT search engine was used, and parameters were specified as follows: fully tryptic specificity, maximum of two missed cleavages, minimum peptide length of 6, fixed carbamidomethylation of cysteine residues (+ 57.02146 Da), variable modifications for oxidation of methionine residues (+ 15.99492 Da), precursor mass tolerance of 15 ppm and a fragment mass tolerance of 0.02 Da for MS2 spectra collected in the Orbitrap. After spectral assignment, peptides were assembled into proteins and were further filtered based on the combined probabilities of their constituent peptides to a final FDR of 1%.

### Reverse transcription-quantitative polymerase chain reaction (RT-qPCR)

The extracted total RNA was incubated with genomic DNA (gDNA) remover at 36 °C for 30 min to remove gDNA. cDNA was synthesized using a cDNA synthesis kit (Takara Bio Inc., Kusatsu, Japan), following the manufacturer’s instructions. SYBR Green Mix (Roche Diagnostics, Basel, Swiss Confederation) and a QuantStudio 3 Real-Time PCR System (Thermo Fisher Scientific, Inc.) were used to perform qPCR according to the manufacturer’s instructions. The reaction conditions of the 20 µL system were: the first reaction at 95 °C for 30 s, followed by 95 °C for 10 s, 60 °C for 30 s, and 72 °C for 10 s, over 45 repetitions. The relative expression levels of the indicated genes were quantified using the 2-ΔΔct method. ACTB was used as an internal control. Primer information is listed in Supplementary Table 1.

### Western blot

RIPA buffer (cat. no. R0010; Solarbio) was used to lyse cells, and protein concentrations were determined with a BCA protein assay kit (Beijing Leagene Biotech Co., Ltd.). Protein samples (25 µg/lane) were separated by SDS-PAGE on 10% gels and transferred onto PVDF membranes. The membranes were blocked by protein-free rapid block buffer (Epizyme Pharmaceutical Biotechnology Co, LTD) for 15 min at room temperature and were incubated overnight at 4˚C with primary antibodies against AGPS (1:500, sc-374201, Santacruze), MDM2 (1:500,sc-965, Santacruze), PMP70(1: 1000, ab3421, Abcam), Flag (1: 1000, 201,126-3A6, ZENBIO), c-Myc (1: 1000, sc-40, Santacruze), TrkA (1:1000,2510,CST), p-TrkA (1:1000,PA5-104,674, ThermoFisher), and β-tubulin (1:1000, ABL1030,Abbkine). Following primary antibody incubation, the membranes were further incubated with Dylight 800-Goat Anti-Rabbit IgG (1:100, A23910 Abbkine) or Dylight 800-Goat Anti-Mouse IgG secondary antibodies (1:100, A23920 Abbkine) at 25˚C for 1 h. The membranes were then scanned by an imaging system (ODYSSEY ® CLx, Gene Company limited), and the optical density was measured using Image Studio Lite (LI-COR Biosciences).

### Co-immunoprecipitation

The cells were lysed on ice for 30 min in 1 mL lysis buffer (Servicebio, G2038), containing protease inhibitor MG132 (Selleck, S2619). After centrifugation at 17,000 g for 30 min, the supernatant was collected, where 100ul was used as the input, and the rest was shaken overnight at 4 °C with protein A + G Agarose IP reagent (Bioworld, BD0048) and 10 ul Anti-Flag antibody (ZENBIO, T201126-3A6). The beads were washed three times with lysis buffer and boiled in 2X SDS loading buffer for 5 min to release the bound proteins from the beads. Western blot was performed to detect the proteins in the IP samples.

### GST pull-down assay

GST pull-down was used to precisely determine the bonding positions between AGPS and MDM2 in vitro.

#### Protein expression and purification

The plasmids were constructed on the pGEX-4 T-1 vector and expressed in the *E. coli* BL21 (DE3) strain. GST parallel genes were transformed in BL21 cells and spread on LB plates with AMP. A colony was selected and grown in 10 ml LB (Amp) overnight at 37℃, then transferred to 500 ml LB and grown at 25℃ until OD600 = 0.6. The colony was then incubated with 0.5 mM IPTG at 16℃ for more than 15 h. The cells were harvested the next day and centrifuged at 3000 rpm for 20 min at 4 °C. The cells were then resuspended with cold PBS and transferred to a 50 ml tube, followed by additional centrifugation and resuspension with lysis buffer (20 ml/l of culture, with 1 mM PMSF and 1 mM cocktail). All the steps were performed at 4℃ on ice. The cells were ultrasonicated 2–3 times and centrifuged at 12,000 rpm for 30 min, before transferring the supernatant into a clean tube. This process was repeated twice to obtain a clear lysate.

#### GST purification

The samples were loaded into pre-clean GST Purification Magbeads (Absin, abs9902) (120 ul of beads for 200 ml culture) and shaken for 2 h. The beads were centrifuged and washed with 10 ml GST clean buffer(BJBiolab, GS4655), supplemented with a PMSF cocktail. Finally, the beads were centrifuged and collected.

#### GST pull-down

The GST beads were mixed and shaken overnight at 4 °C with a protein supernatant containing overexpressed Myc-MDM2 or Flag-AGPS. The beads were washed three times with elution buffer (BJBiolab, GS4654) and boiled in 2X SDS loading buffer for 5 min to release the bound protein from the beads. Western blot was performed to detect the proteins in the GST pull-down samples.

### Immunohistochemistry

The prepared tissue paraffin sections were soaked in xylene solution for 10 min, then soaked in 100%, 95%, 80%, and 70% ethanol solutions for 2 min for dehydration, and finally washed with distilled water for 5 min. Then destroy the endogenous peroxidase activity by methanol and 30% H_2_O_2_.The samples were then washed with PBS (3 min × 3) and sealed with serum at room temperature for 30 min. Paraffin-embedded tissues were sliced into 5-μm sections and observed for AGPS (1:500, sc-374201, Santacruze) and p-TrkA (1:1000, PA5-104674, ThermoFisher), expression after immunohistochemistry staining. These sections were dyed with diluted anti-AGPS and anti-p-TrkA antibody and incubated at 4 °C overnight; then, they were washed three times with fresh phosphate-buffered saline (PBS) solutions and immediately incubated with biotin-labeled second antibody for immunohistochemistry (GB23303; Servicebio, Wuhan, China) for 50 min at room temperature. The positive cells were monitored using diaminobenzidine solution (K5007; DAKO, Santa Clara, CA, USA) by direct viewing. The processed slides were observed using a light microscope (Leica DM2700). Every immunohistochemistry result was read blindly and independently by two senior pathologists. The study design was approved by the Ethics Committee of the First Affiliated Hospital of Zhengzhou University (2022-KY-0976).

### Immunofluorescence staining

Wash cells on slides once with 1X PBS without Ca and Mg and fix cells in 2%paraformaldehyde in PBS for 20 min. Then wash cells with PBS for three times, each time for 5 min. Then add permeabilize solution (0.2% triton) for 15 min. Wash cells with PBS for three times, each time for 5 min and then block the slides with blocking buffer (5% glycerol,5% of goat serum in 1X PBS). Then, diluted primary antibodies were added and incubated overnight at 4℃. After washing three times, the corresponding secondary antibody was added and incubated for approximately 1 h. DAPI reagent was added, and the slices were incubated in the dark for 5 min. After washing, the slides were fixed with an anti-fluorescence quenching agent and observed under a fluorescence microscope.

### Colony formation assay

The proliferative ability of the cells was analyzed using a colony formation assay. Approximately 3000 cells were cultured with 2 ml MEM media containing 10% FBS in each well of a 6-well plate. The cells were cultured for 7 days and fixed with paraformaldehyde for 30 min. The cell colonies were then stained with a crystal violet staining solution for 2 h. A microscope (Leica DM IRB, Wetzlar, Germany) was used to capture the images.

### Transmission electron microscopy

Cells were centrifuged to agglomerates, 2.5% glutaraldehyde, phosphate buffer preparation fixed for 2 h, rinsed three times with 0.1 M phosphate rinse solution for 15 min each, 1% osmium acid fixed for 2–3 h; rinsed three times with 0.1 M phosphate rinse solution for 15 min each. After dehydration with 50% ethanol, 70% ethanol, and 90% ethanol, respectively; acetone-embedded and fixed, the sections were sectioned (70 nm) (Leica EM KMR3), double-stained with 3% uranyl acetate-lead citrate and observed using transmission electron microscopy (JEM-1230), and photographed.

### CCK8 assay

Cells were plated in 96-well plates at 3 × 10^3^ cells/well. The cell media in each well was then replaced with 100 μl new media and 10 μl CCK-8 reagent (Dojindo, Tokyo, Japan) and incubated for 1 h at the indicated time points (0, 1-, 2-, 3-, and 4-days post-transfection). Optical density at 450 nm (OD450) was measured using a DNM-9606 microplate reader (Perlong, Beijing, China).

### Fe^2+^ assay

Cells in a 10 cm petri dish were collected and re-suspended in 1 ml cold PBS. After repeated blowing with a pipette gun, the supernatant was removed. The supernatant was stirred with a vortex mixer for 10 s and centrifuged at 14,000 rpm for 4 min. Then the Fe^2+^ assay was performed with the Iron Assay Kit (ab83366, Abcam, USA) as per the manufacturer’s instructions.

### MDA assay

A micro-malondialdehyde (MDA) assay kit (Solarbio, BC0025) was used to detect the level of lipid oxidation in cell membranes. The cells were collected in a centrifuge tube, and the supernatant was discarded after centrifugation. Reagent I (1 ml) was added per 5 million cells. Ultrasonic waves (power, 20%; ultrasonic waves, 3 s; interval, 10 s; repetitions, 30 times) were used to break the cells before centrifugation at 8000 × g for 10 min at 4 °C. The supernatant was then collected and placed on ice before testing. The spectrophotometer or microplate reader was preheated for more than 30 min and the wavelength was set to 415 nm. The samples and Reagents I, II, and III were then mixed sequentially. After the mixture was kept warm in a water bath at 100 °C for 60 min (capped tightly to prevent water loss), it was cooled in an ice bath at room temperature and centrifuged at 10,000 × g for 10 min. A 200-μL aliquot of the supernatant was pipetted into a 96-well plate to measure the absorbance at 450, 532, and 600 nm to determine the ΔA, from which the MDA content was calculated.

### Molecular docking study

The docking study of the AGPS and MDM2 (protein–protein) interaction was performed according to the ZDOCK module of Discovery Studio 3.0, and the docking was performed using rigid docking. The simulation of the phosphorylation concept of AGPS at the Y451 was performed using Amber18, and the most stable structure was selected for molecular docking with MDM2 again.

### In vivo tumor xenograft model

All SCID mice were purchased from Sipeifu Company (Beijing, China). Approximately 1 × 10^6^ cells were suspended with the Matrigel (Becton, Dickinson and Company, USA) in a 1:1 ratio, and then extracted with a 1 ml syringe. The needle was inserted into the groin at 45° before injection. After pressing the pinhole lightly with the left index finger for about 1 min, the mice were put back into the feeding cage. After 33 days, the mice were sacrificed, and the tumor masses were removed for photographs. The mice experiments were approved by the Ethics Committee of the First Affiliated Hospital of Zhengzhou University (ZZU-2022-KY-0976).

### Organoid culture

Mince human prostate cancer tissues (collected from the first affiliated hospital of Zhengzhou University) in small pieces (1 – 5 mm^3^) in a 10 cm culture using a scalpel. The tissue should be digested overnight in a solution containing 5 mg/ml Collagenase II and 10 µM Y-27632, using a 15 ml tube on a shaking platform at a temperature of 37 °C. It is recommended to use 1 ml of the 5 mg/ml Collagenase II solution for approximately every 50 mg of minced tissue. Following digestion, the tissue should be washed once by adding enough F12K to reach a total volume of 10 ml. The resulting mixture should then be centrifuged at 200 g for 5 min at a temperature of 4 °C.The pellet should be resuspended in 1 ml of TrypLE containing 10 µM Y-27632 and digested for approximately 15 min at a temperature of 37 °C. Following this, the pellet should be washed once by adding enough F12 medium to reach a total volume of 10 ml, and then centrifuged at a speed of 200 g for 5 min at a temperature of 4 °C. The supernatant should be aspirated and the digested tissue should be placed in ice-cold Matrigel. To ensure proper mixing, the tissue should be pipetted up and down 5–10 times. The cells should then be counted using a hemocytometer, and approximately 20,000 cells should be plated in a 40 µl drop in the center of one well of a 24-well dish. The dish should be placed in a 37 °C incubator for 15 min to allow the Matrigel to solidify. Carefully transfer 500 µl of pre-warmed (37 °C) human prostate culture medium supplemented with 10 µM Y-27632 into each well using a pipette. Replace the medium every 2 – 3 days. After a period of 7 days, eliminate the presence of Y-27632 from the medium.

### Statistical analysis

All data were analyzed using GraphPad Prism 9.0 (LaJolla, USA) and expressed as the mean ± SD. Student's t-test or ANOVA was used to analyze the groups unless otherwise indicated. *P* values < 0.05 indicated statistical significance.

## Results

### AGPS plays a key role in the progression and prognosis of prostate cancer

**Fig. 1:**
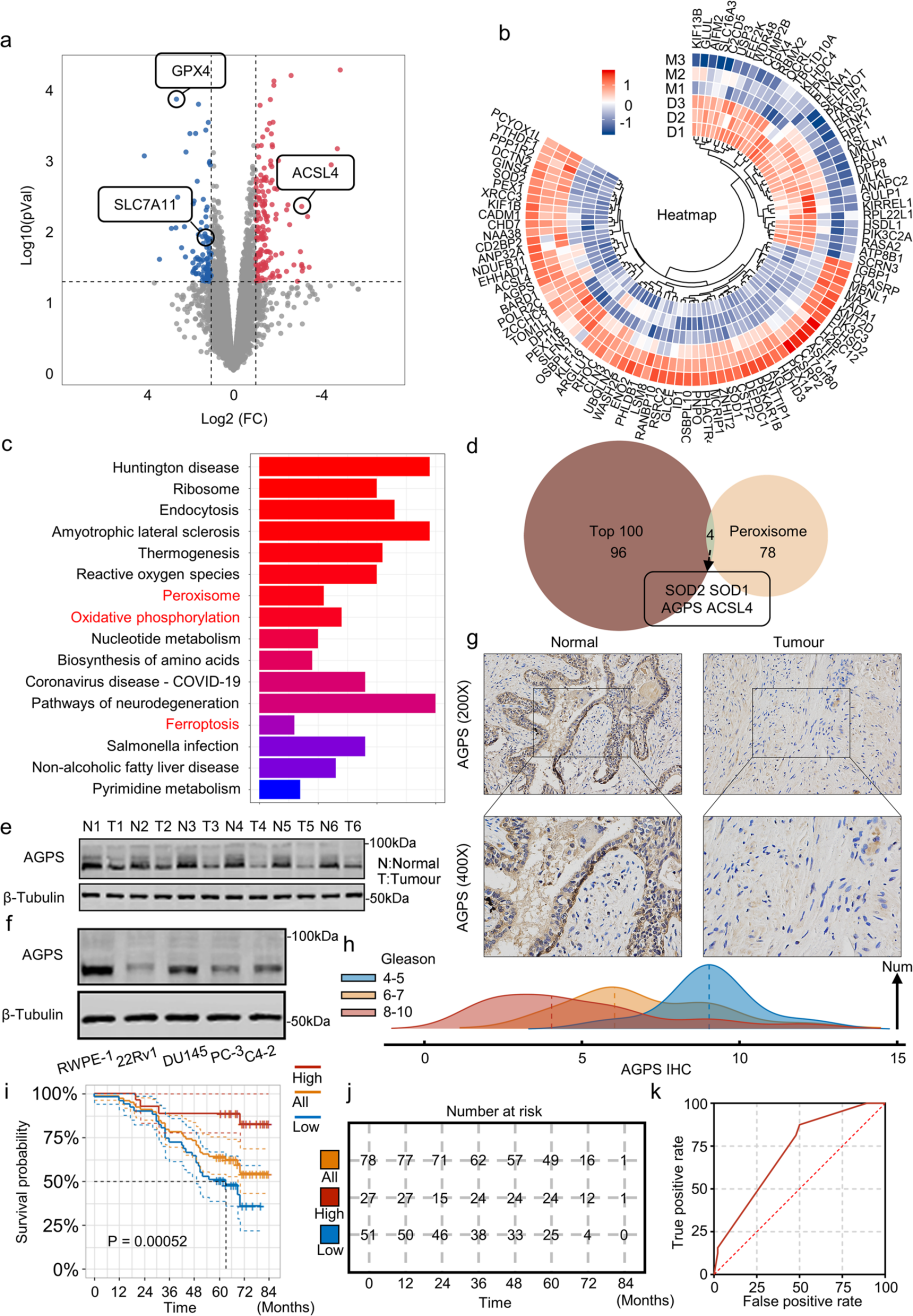
AGPS plays a key role in the progression and prognosis of prostate cancer.** a** The Volcano map of Lable-Free MS after ML210 treatment. **b** The heatmap of top 100 upregulated and downregulated proteins after ML210 treatment. **c** KEGG analysis of changes proteins after ML210 treatment. **d** The overlapping Venn diagrams of peroxisome genes and the top 100 upregulated and downregulated genes after ML210 treatment. **e** AGPS protein expression in PCa tissues and normal tissues. f. AGPS protein expression in different PCa cell lines. **g** AGPS IHC staining in PCa and normal tissues. **h** Statistics of AGPS IHC staining. **i**, **j** Survival percentage of high or low AGPS expression groups based on IHC score. **k** ROC curves demonstrate the sensitivity and specificity of AGPS as a marker for PCa prediction

Ferroptosis is a type of necrosis that is driven by iron-dependent lipid peroxidation. It is closely associated with a variety of biological scenarios, including cell development, aging, immunity, and cancer. Besides that, ferroptosis affects a wide range of cellular functions such as metabolism and ROS production, but its mechanism in PCa remains unclear. To investigate the role of ferroptosis in PCa, we performed Label-Free Mass spectrometry after treating DU145 cells with ferroptosis inducer-ML210(10uM for 8 h) and identified the potential targets. Our data displayed 206 upregulated proteins and 150 downregulated proteins (LogFC > 2, *P* < 0.05) (Fig. 1a). Among these, the inhibitory effects of GPX4 and SLC7A11 on lipid peroxidation are widely acknowledged, leading to their ability to regulate ferroptosis negatively [18, 19]. Conversely, ACSL4 promotes ferroptosis through the initiation of phospholipid peroxidation [20]. According to our results, GPX4 and SLC7A11 were significantly downregulated, while ACSL4 were significantly upregulated, which confirmed the effectiveness of ML210 treatment. We selected the top 100 genes according to expression differences (LogFC > 2, *P* < 0.05) (Fig. 1b). Then we performed the KEGG analysis (Fig. 1c) and GO analysis (Supplementary Fig. 1a). The KEGG analysis reported that the peroxisome synthesis-related functions which involved in ferroptosis process were significantly activated after ML210 treatment. So, we selected intersections with our Lable-Free Mass spectrometry of the top 100 differentially expressed proteins and 84 peroxisome related proteins and identified 4 related proteins: SOD2, AGPS, ACSL4 and SOD1 (Fig. 1d). Among them, the role of SOD2, ACSL4, and SOD1 in ferroptosis has been well-reported, but the role of AGPS in ferroptosis remains unclear. Therefore, we selected AGPS for subsequent investigation.

The TCGA data indicated that AGPS was significantly downregulated in PCa tissues (Supplementary Fig. 1b) and the expression differences were more pronounced at higher T stages (Supplementary Fig. 1c). And we also found the different expression of AGPS in different prostate cancer cell lines from the CCLE database (https://sites.broadinstitute.org/ccle/) (Supplementary Fig. 1d). But the mRNA expression seems to be no significantly different in different Gleason scores (Supplementary Fig. 1e). The comparison of 20 pairs of PCa tumor and normal tissues demonstrated that AGPS mRNA expression was not very significantly different between the tumor tissues and normal tissues (Supplementary Fig. 1f), which coordinated with the results in the cell lines (Fig. 1g). Then, we detected the protein levels of AGPS between prostate tissues and the normal tissues, result suggested that AGPS protein expression decreased in prostate cancer compared with the normal tissues (Fig. 1e). Additionally, we examined the protein expression of AGPS in different prostate cancer cell lines by Western blot (Fig. 1f), which lead to the same results.

Subsequently, we selected 89 pairs of PCa tumor and normal tissues for IHC staining and found that the positive rate of AGPS was significantly lower in PCa tumors than in normal tissues (Fig. 1g and h). We then performed a prognostic analysis based on the expression of AGPS in the 89 pairs of cases collected and found a significant association between AGPS and poor prognosis in PCa patients (Fig. 1i, j and k). These results suggest that AGPS is downregulated in PCa tissues and cells. Aditionally, AGPS is associated with better prognosis in PCa patients.

### AGPS inhibits the proliferation of prostate cancer cells by promoting the formation of peroxisome and ferroptosis

**Fig. 2:**
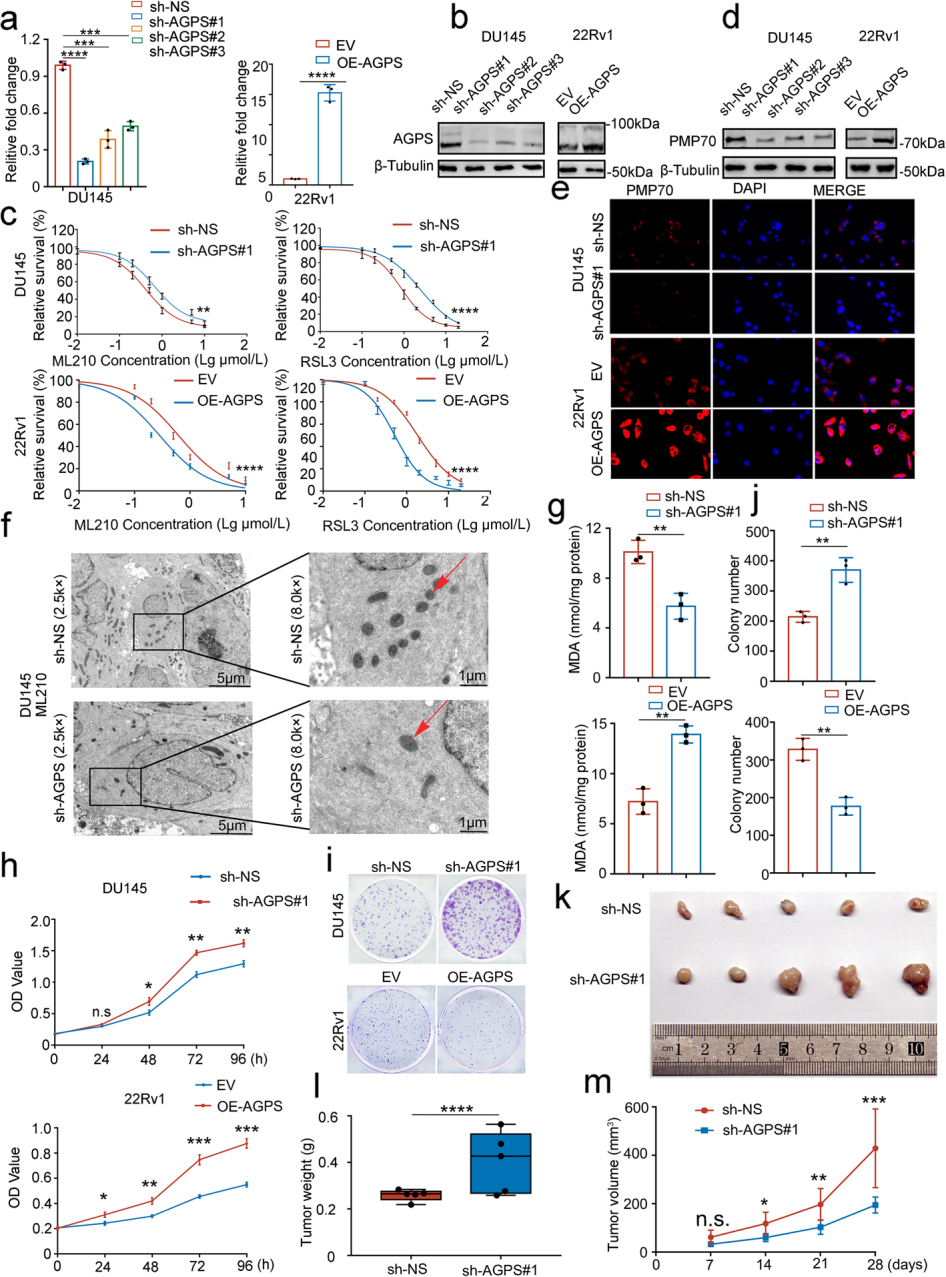
AGPS inhibits the proliferation of prostate cancer cells by promoting the promotion of peroxisome production and ferroptosis.** a** AGPS expression in mRNA after transfection with three shRNAs in DU145 and over expression in 22Rv1 cells. * *P* < 0.05, ** *P* < 0.01, *** *P* < 0.001. **b** AGPS expression in proteins after transfection with three shRNAs in DU145 and over expression in 22Rv1 cells. **c**. IC50 values of ML210 and RSL3 in DU145 cells after AGPS knockdown and in 22Rv1 cells after AGPS over expression. ** *P* < 0.01, *** *P* < 0.001, **** *P* < 0.0001. **d** PMP70 protein expression after AGPS knockdown with shRNA in DU145 cells and over expression in 22Rv1 cells. **e** PMP70 protein immunofluorescence staining after AGPS knockdown with shRNA in DU145 cells and over expression in 22Rv1 cells. **f** PCa cells were observed by TEM when induced with ML210 after AGPS knockdown. **g** MDA levels after AGPS knockdown with shRNA in DU145 cells and over expression in 22Rv1 cells. ** *P* < 0.01. **h** CCK8 assay on the OD value in 6-well plates after AGPS knockdown with shRNA in DU145 cells and over expression in 22Rv1 cells. n.s., no significance, * *P* < 0.05, ** *P* < 0.01. **i** and **j** Colony formation assay on colony numbers of the DU145 cells after AGPS knockdown with shRNA and 22Rv1 cell with AGPS over expression. ** *P* < 0.01. **k** PCa cells after AGPS knockdown were injected subcutaneously into the right flank of mice. Xenograft growth was measured every other day for 28 days. Tumors in each group at day 28 were harvested and photographed. **l** Tumor weight in each group. Data represented as mean ± SD (*n* = 5). **** *P* < 0.0001. **m** Tumor volume at each time point. Data represented as mean ± SD (*n* = 5). n.s., no significance; *P < 0.01, **P < 0.01, ***P < 0.001

In order to investigate whether AGPS acts through the ferroptosis in prostate cancer, we conducted colony formation experiments as an initial approach. Specifically, we employed overexpression of AGPS in 22Rv1 cells, which exhibit relatively low AGPS expression levels. Subsequently, we treated the cells with a combination of cellular autophagy inhibitor (CQ,25 μM), ferroptosis inhibitor (Ferrostatin-1,1 μM), and apoptosis inhibitor (Z-VAD-FMK,40 μM), commencing drug administration on the third day. On the seventh day, we assessed the clonal growth of the cells and found that the addition of the ferroptosis inhibitor alone resulted in a significant restoration of cell growth (Supplementary Fig. 2a and b). To investigate the specific role of AGPS in PCa through ferroptosis, we selected three cell lines (i.e., DU145, 22Rv1 and PC-3) for further investigations. DU145 and PC-3 reported relatively higher AGPS expression, whereas 22Rv1 reported a lower expression (Fig. 1f). We knocked down AGPS expression with three different lentivirus packaging short hairpin RNA (shRNA) in DU145 and PC-3 cell lines and over-expressed AGPS in 22Rv1 cell line (Fig. 2a and b, supplementary Fig. 2e, f). Then we examined the sensitivity of PCa cells to ferroptosis inducers. We found that the IC50 of both DU145 and PC-3 cells to ML210 and RSL3 was remarkably decreased after AGPS knockdown (Fig. 2c, supplementary Fig. 2g) as well as increased significantly after AGPS over-expression in 22Rv1 cells, indicating a correlation between AGPS expression and sensitivity to ferroptosis inducers in PCa. We also noticed the expression of peroxisome membrane protein 70 (PMP70) significantly reduced after AGPS knockdown and increased after AGPS over-expression, which suggested a variation in peroxisome production (Fig. 2d and e, Supplementary Fig. 2c, i,j and k). But there were no significant changes in Fe^2+^ (Supplementary Fig. 2d and h). Our findings reported that when induced with ML210, mitochondria with increased membrane density and atrophy mitochondria reduced after AGPS knockdown through TEM (Transmission electron microscopy) (Fig. 2f, Supplementary Fig. 2l). Similarly, malondialdehyde (MDA), the main product of ferroptosis, was greatly reduced after AGPS knockdown, meanwhile, increased after AGPS over expression (Fig. 2g and Supplementary Fig. 2m). These verified our hypothesis that AGPS could promote the ferroptosis process.

The findings derived from the CCK8 and clone formation assays further substantiate that the downregulation of AGPS elicits a stimulatory effect on the proliferation of PCa cells, while the overexpression of AGPS exerts an inhibitory influence on their proliferation (Fig. 2h-j, Supplementary Fig. 2n-p). To further verify the role of AGPS in PCa, we performed a subcutaneous transplantation tumor model using severely immunodeficient mice and found that the tumor progressed by AGPS knockdown (Fig. 2k-m).

Overall, the above results suggested that AGPS can contribute to the development of ferroptosis by promoting peroxisome formation and inhibiting the proliferation of PCa cells.

### MDM2 ubiquitinates and modifies AGPS and promotes its degradation

**Fig. 3:**
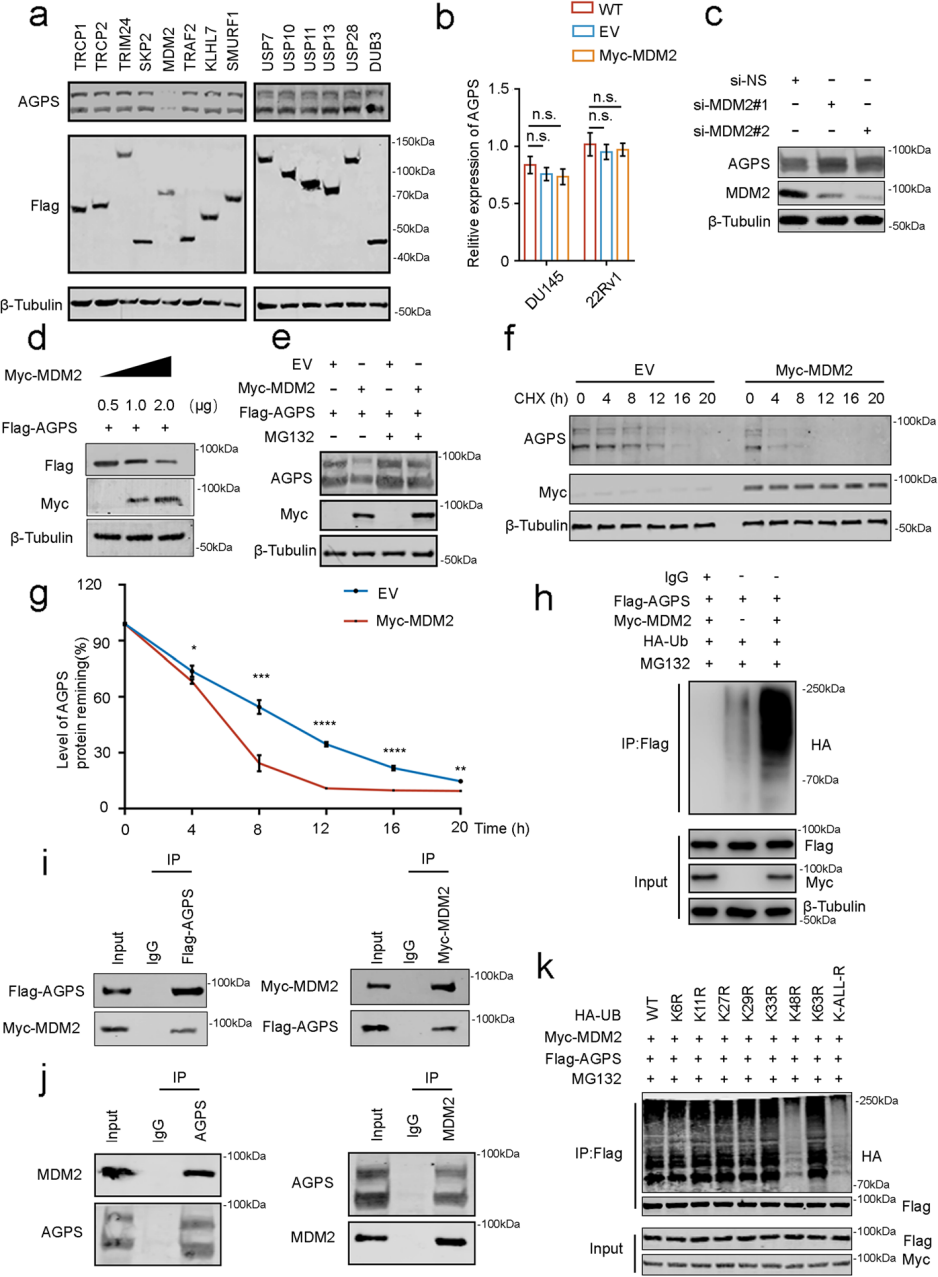
MDM2 ubiquitinates and modifies AGPS and promotes its degradation. **a** Western blot of AGPS expression after ectopic transfection with different E3 or DUBs. **b** qPCR of the AGPS expression after ectopic transfection with MDM2. **c** AGPS expression after MDM2 knockdown. n.s. no significance. **d** Western blot of the AGPS expression after ectopic transfection with different doses of MDM2. **e** Western blot of the AGPS expression after ectopic transfection with MDM2 and treatment with or without MG132 (20 μM) for 8 h. **f** Western blot analysis of AGPS protein expression in PC-3 cells after AGPS knockdown followed by treatment with cycloheximide (CHX) (50 μg/mL) for 0-, 4-, 8-, 12-, 16-, or 20- h. **g** The protein bands were quantified and normalized to the band intensity at the 0-h time point. * *P* < 0.05, ** *P* < 0.01, *** *P* < 0.001, **** *P* < 0.0001. **h** Ubiquitination experiment on the changes in AGPS ubiquitination levels after ectopic transfection with MDM2 and treatment with MG132 (20 μM) for 8 h. **i**,** j** Ectopia and endogenic MDM2 and AGPS binding. **k** Ubiquitination experiment on the changes in AGPS ubiquitination levels after ectopic transfection with MDM2 and different lysis mutant-type of UB

To understand the specific mechanisms involved in the lower expression of AGPS in PCa, we treated PCa cells with the proteasome inhibitor MG132 and the lysosomal inhibitor hydroxychloroquine. We found that AGPS expression was significantly upregulated after MG132 treatment, but no significant changes were observed in hydroxychloroquine-treated cells (Supplementary Fig. 3a). Thus, we speculated that the degradation of AGPS protein might be related to the proteasome pathway. We established the interaction between E3 ligases and AGPS using the Ubibrowser (http://ubibrowser.ncpsb.org/) to predict the possible E3 ligases of AGPS and used the Biogird database (https://thebiogrid.org/) to identify the potentially interacting proteins with AGPS. The combined analysis indicated that MDM2 proteins may interact with and regulate AGPS proteins (Supplementary Fig. 3b). Meanwhile, we transfected different E3 ligases and DUBs in PCa cells, and the level of AGPS protein was significantly decreased in the MDM2 ectopically transfected cells (Fig. 3a), which was consistent with our predictions from the analysis. We then ectopically transfect MDM2 but found no remarkable changes in AGPS at the transcriptional level (Fig. 3b). Subsequently, we found that MDM2 knockdown significantly increased the AGPS expression in a dose-dependent manner (Fig. 3c and d), and the process could be hindered by MG132 treatment (Fig. 3e). This result suggested that MDM2 affects AGPS stability via the ubiquitin–proteasome pathway. Meanwhile, the half-life of AGPS significantly decreased after ectopic expression of MDM2, which confirmed that MDM2 could degrade AGPS (Fig. 3f and g).

Next, we examined the ubiquitination level of AGPS after ectopic transfection with MDM2 and found that MDM2 overexpression increased the ubiquitination level of AGPS, indicating that MDM2 could alter the ubiquitination level of AGPS (Fig. 3h). On a molecular level, we examined the possible interactions between MDM2 and AGPS. The co-immunoprecipitation (co-IP) assay confirmed that both ectopically expressed and endogenous AGPS and MDM2 interacted with each other (Fig. 3i and j). To investigate the type of ubiquitin linkage on AGPS, we mutated the lysine (K) and arginine (R) residues on ubiquitin and evaluated their effects on AGPS ubiquitination after MDM2 transfection. Results demonstrated that K6R-, K11R-, K27R-, K29R-, K33R- and K63R-ubiquitin could trigger MDM2-mediated AGPS ubiquitination, but the lysine 48 (K48) R-ubiquitin largely inhibited the ubiquitination of AGPS (Fig. 3k). These results also support the notion that MDM2 could mediate AGPS ubiquitination and degradation. To further verify our conclusion, we overexpressed AGPS with or without MDM2 overexpression (Supplementary Fig. 3c). As expected, we observed that mitochondria with increased membrane density and atrophy mitochondria increased significantly, and the phenomenon was reversed by MDM2 overexpression (Supplementary Fig. 3d and e). Similarly, the level of MDA was reversed by the overexpression of MDM2 (Supplementary Fig. 3f). On the contrary, the CCK8 and clone formation assays indicated that AGPS overexpression inhibited tumor cell growth, and concomitant overexpression of MDM2 resulted in significantly higher PCa cell activity as compared to the experimental group overexpressing AGPS alone (Supplementary Fig. 3g-i).

Collectively, our experiments revealed that AGPS, a new substrate for MDM2, can be ubiquitinated and degraded by E3 ligase MDM2.

### AGPS binds to MDM2 at the C-terminal amino acid sequence of the F443-F455 segment

**Fig. 4:**
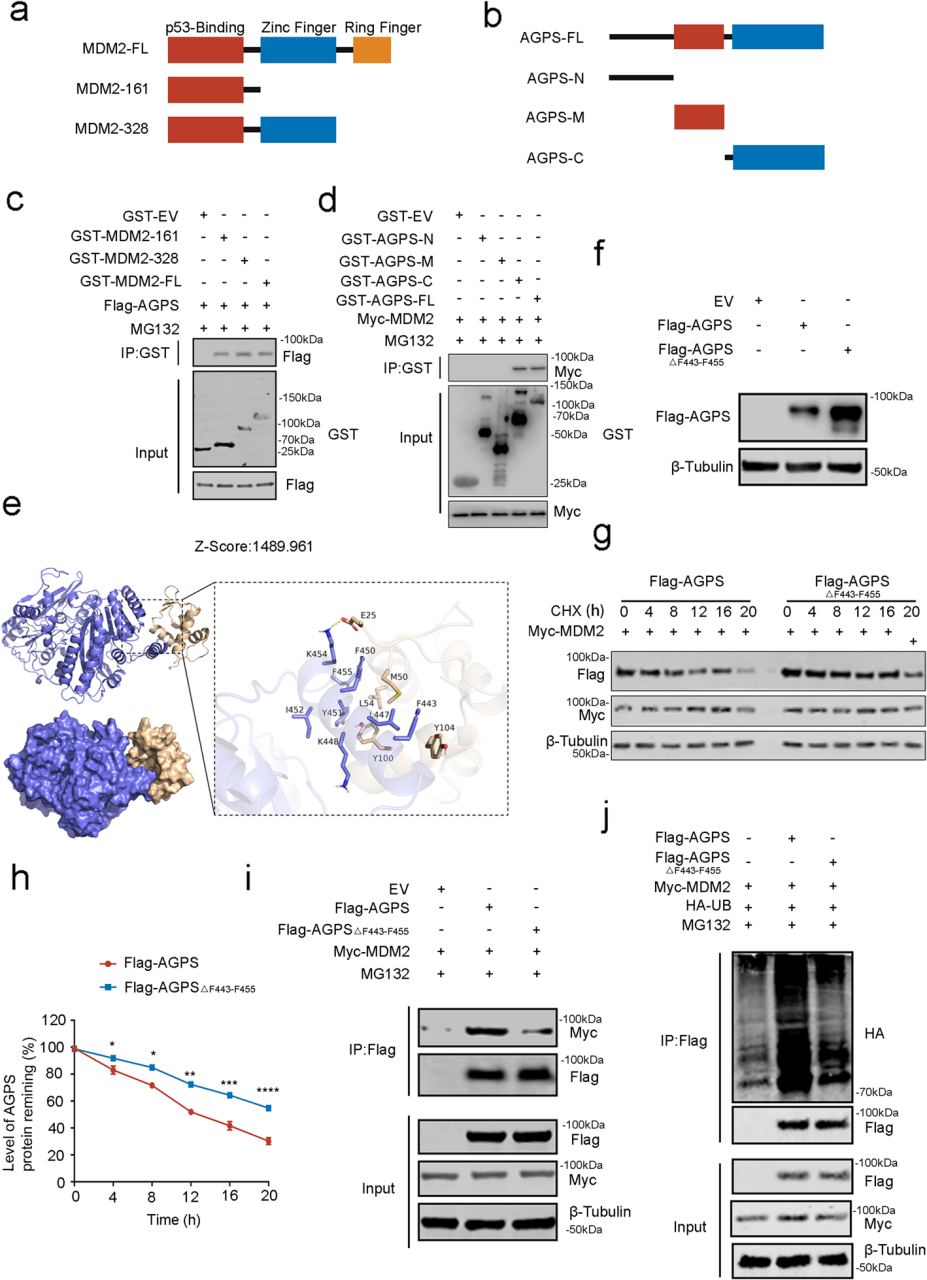
AGPS binds to MDM2 at the C-terminal amino acid sequence of the F443-F455 segment. Schematic diagrams depicting a set of recombinant protein constructs for **a** GST-MDM2 and **b** GST- AGPS. **c** Western blot analysis of AGPS proteins in DU145 whole-cell lysate pulled down by GST or GST-MDM2 recombinant proteins. **d** Western blot analysis of MDM2 proteins in DU145 whole-cell lysate pulled down by GST or GST-AGPS recombinant proteins. **e** Schematic diagrams depicting the potential binding motif of AGPS with MDM2 protein, molecular dock with AGPS (AlphaFold: AF-O00116-F1) and MDM2(6KZU).** f** Western blot analysis of ectopically expressed Flag-tagged AGPS protein in DU145 cells transfected with wild-type or mutant AGPS. **g** Western blot analysis of AGPS protein expression in DU145 cells after ectopically expressed wild type or mutant AGPS followed by treatment of CHX (50 μg/mL) for 0-, 4-, 8-, 12-, 16-, or 20- h. **h** Protein bands were quantified and normalized to the band intensity at the 0-h time point. * *P* < 0.05, ** *P* < 0.01, *** *P* < 0.001. **i** Co-IP analysis of binding of Flag-tagged WT AGPS or mutant AGPS with ectopically expressed Myc-tagged MDM2 in DU145 cells treated with MG132 (20 μM) for 8 h. **j** Western blot analysis of ubiquitination levels in ectopically expressed Flag-tagged WT AGPS or mutant AGPS with ectopically expressed Myc-tagged MDM2 in DU145 cells treated with MG132 (20 μM) for 8 h

To verify the specific binding motifs of AGPS and MDM2, we generated GST recombinant proteins for AGPS and MDM2 (Fig. 4a and b). Except for the full length, the two truncated MDM2 recombinant proteins contained the domains of p53 binding domain or not (Fig. 4a). GST pull-down assay reported that the p53 binding domain could bind to AGPS (Fig. 4c), while the C-terminus of AGPS could bind to the C-terminus of MDM2 (Fig. 4d). To verify this, we simulated the spatial structures of AGPS and MDM2 proteins using the Z-DOCK software and subsequently performed molecular docking for the two proteins. The high Z-score suggests a strong binding of the two proteins (Fig. 4e). Moreover, the result of the molecular docking revealed the amino acids in the C-terminus of AGPS that could be specific binding sites for MDM2. This included F443, L447, K448, F450, Y451, I452, K454, and F455.

To validate the binding sites, we constructed the AGPS delete mutant of AGPS F443-F455 region and transfected the mutant AGPS into PCa cells. We found that the protein expression and half-life of mutated AGPS were significantly higher or longer than wild-type AGPS (Fig. 4f–h). In contrast, there seems less binding of mutated AGPS with MDM2and the level of ubiquitination of AGPS by MDM2 also significantly reduced after AGPS mutation (Fig. 4i and j). These results suggest that the binding of mutated AGPS and MDM2 was greatly reduced, which resulted in a decline in ubiquitination and degradation of AGPS. In summary, these experimental results identified the specific binding sites of AGPS and MDM2 and revealed the regulatory relationship between them.

### Phosphorylation modification of the Y451 site leads to the accumulation of AGPS in prostate cancer cells

**Fig. 5:**
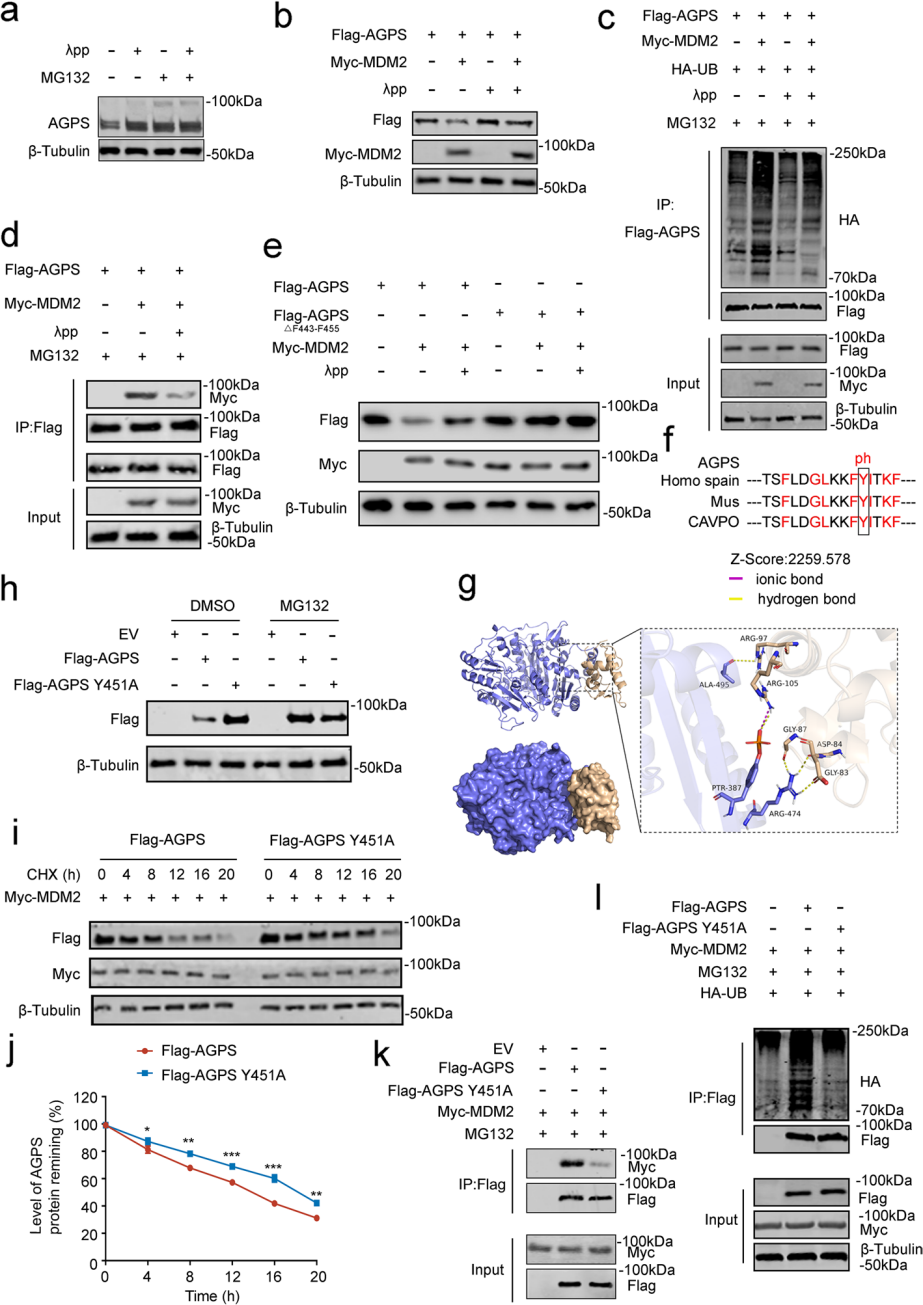
Phosphorylation modification of the Y451 site leads to the accumulation of AGPS in prostate cancer cells.** a** Western blot analysis of AGPS expression after MG132 (20 μM), λpp(1U/ml,2 h), or a combination. **b** Western blot analysis of ectopically expressed Flag-tagged AGPS protein in DU145 cells transfected with AGPS followed by treatment with λpp(1U/ml,2 h). **c** Western blot analysis of ubiquitination levels in ectopically expressed Flag-tagged AGPS and ectopically expressed Myc-tagged MDM2 in DU145 cells treated with MG132 (20 μM) for 8 h or λpp (1U/ml,2 h). **d** Co-IP analysis of binding of Flag-tagged AGPS with ectopically expressed Myc-tagged MDM2 in DU145 cells treated with λpp(1U/ml,2 h). **e** Western blot analysis of ectopically expressed Flag-tagged AGPS protein in DU145 cells transfected with Flag-tagged WT AGPS or mutant AGPS with or without λpp (1U/ml,2 h) treatment. **f** Alignment of amino acid sequences of the F443-F455 region of the AGPS protein. **g** Schematic diagrams depicting the potential binding and Z score of ADPS and MDM2 protein binding after AGPS Y451 site mutation to A. **h** Western blot analysis of ectopically expressed Flag-tagged AGPS protein in DU145 cells transfected with wild-type AGPS or AGPS Y451A mutant followed treatment with MG132 (20 μM) for 8 h. **i** Western blot analysis of AGPS protein expression in DU145 cells after ectopically expressed wild-type or mutant AGPS followed by treatment with CHX (50 μg/mL) for 0-, 4-, 8-, 12-, 16-, or 20- h. **j** Protein bands were quantified and normalized to the band intensity at the 0-h time point. * *P* < 0.05, ** *P* < 0.01. **k** Co-IP analysis of binding of Flag-tagged WT AGPS or mutant AGPS with ectopically expressed Myc-tagged MDM2 in DU145 cells treated with MG132 (20 μM) for 8 h. **l** Western blot analysis of ubiquitination levels in ectopically expressed Flag-tagged WT AGPS or mutant AGPS with ectopically expressed Myc-tagged MDM2 in DU145 cells treated with MG132 (20 μM) for 8 h

The expression of MDM2 did not significantly change in PCa (Supplementary Fig. 4a), but its substrate, AGPS, was significantly downregulated. Interestingly, a study previously reported that the ubiquitination modification and degradation function of MDM2 for AR was affected by phosphorylation modification [21]. The research proposed the function of MDM2 ubiquitination modification in a phosphorylation-dependent manner. To validate that the phosphorylation of AGPS could affect the binding of AGPS and MDM2 and lead to a change in AGPS expression, we first treated the cells with lambda protein phosphatase (λpp) to eliminate the function of phosphatase. We found that the expression of AGPS increased after λpp treatment, and this effect was removed after combination treatment with MG132 (Fig. 5a). Subsequently, we transfected both AGPS and MDM2 and found MDM2 can no longer degrade AGPS after λpp treatment (Fig. 5b). Likewise, the level of ubiquitination modification of AGPS by MDM2 significantly decreased after λpp treatment (Fig. 5c). These results suggested that the ubiquitination regulation of AGPS might be influenced by the phosphorylation of AGPS.

Additionally, Co-IP results confirm that the binding of AGPS and MDM2 was affected by λpp (Fig. 5d). We transfected AGPS△F443-F455 and wild-type AGPS in PCa cells and treated the cells with λpp. The result indicated λpp only affected the wild-type AGPS and not the mutated AGPS (Fig. 5e), which verified that AGPS might be modified by phosphorylation at the binding site with MDM2, thus affecting the binding of the two proteins. Among these binding sites, we found that Y451 could be modified by phosphorylation and is highly conserved in evolution (Fig. 5f). Consequently, we speculated that the phosphorylation modification of Y451 was the primary cause. To confirm this speculation, we used the Z-DOCK software to simulate the spatial conformation of AGPS after the phosphorylation of the Y451 site and used this model to dock again with MDM2. Result showed that the binding of the two proteins was greatly increased after docking, and the Z-score significantly increased (Fig. 5g), which indicated that the phosphorylation of Y451 promoted the binding between AGPS and MDM2. To verify this, we constructed an Y451-mutated AGPS and found that the expression and half-life of AGPS significantly increased (Fig. 5h-j). Similarly, the level of AGPS ubiquitination was significantly reduced after simultaneous overexpression of MDM2 after the Y451 mutation (Fig. 5l). Co-IP experiments revealed that the binding of AGPS to MDM2 was significantly weakened after mutation at Y451 (Fig. 5k), which explained the reduced ubiquitination level and protein accumulation of AGPS. Our findings confirmed that AGPS can be phosphorylated at Y451, thus enhancing its binding to MDM2 and AGPS accumulation.

### TrkA promotes the ubiquitinated degradation of AGPS by MDM2 by modifying the phosphorylation of AGPS

**Fig. 6:**
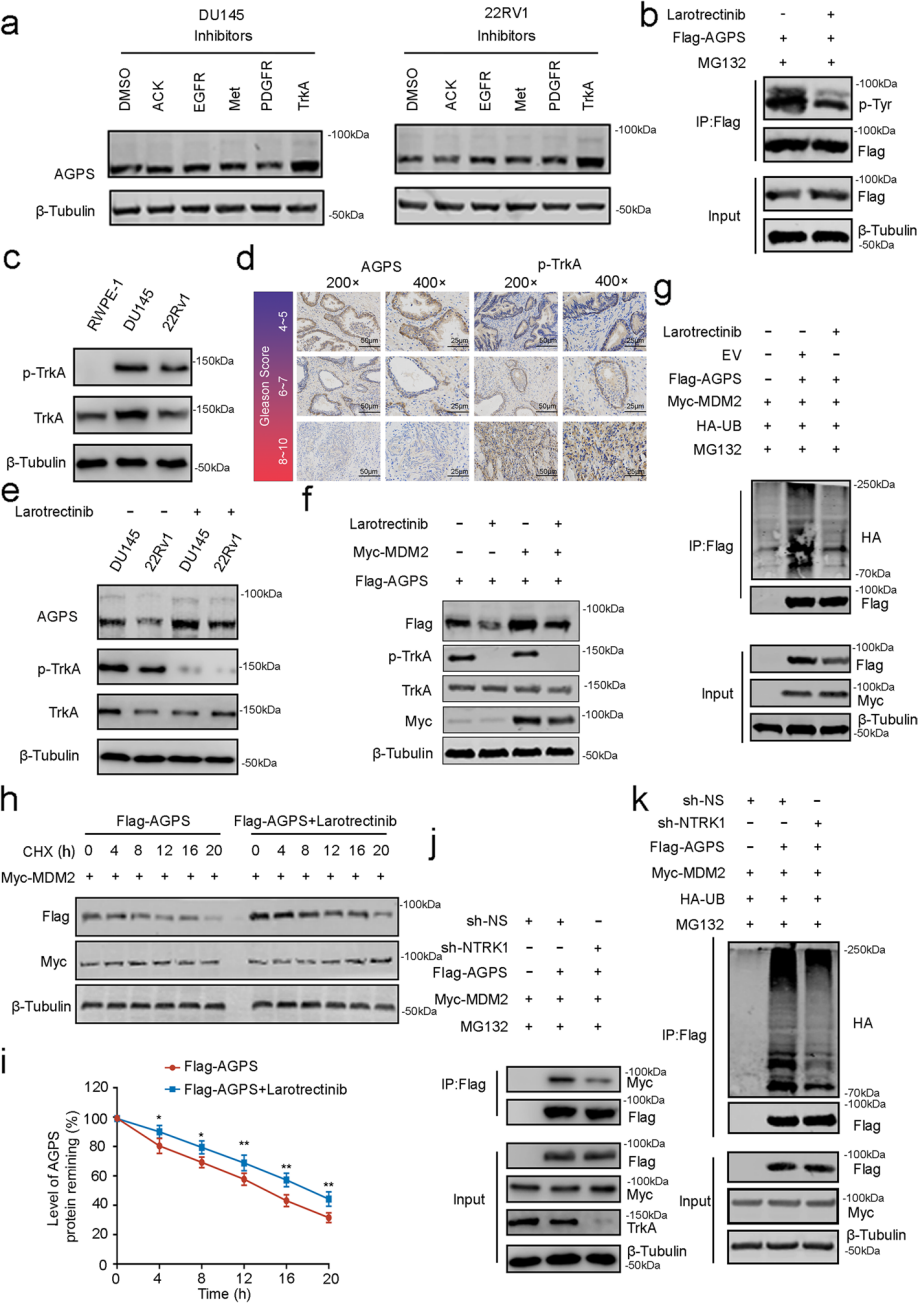
TrkA promotes the ubiquitinated degradation of AGPS by MDM2 by modifying the phosphorylation of AGPS. **a** Western blot analysis of endogenous AGPS proteins in DU145 and 22Rv1 cells followed by treatment with different phosphatase inhibitors (Merestinib, 10 μM for 24 h; AG1295,10 μM for 2 h; AIM-100, 10 μM overnight; Dacomitinib, 2 μM for 24 h; Larotrectinib, 20 nM overnight). **b** Co-IP analysis of the phosphorylation levels of Flag-tagged AGPS in DU145 cells after being treated with MG132 (20 μM) for 8 h and Larotrectinib. **c** Western blot analysis of TrkA and p-TrkA expression in DU145, 22Rv1 and RWPE-1 cells. **d** Analysis of correlation between IHC staining of AGPS and p-TrkA proteins in different Gleason score PCa patient specimens. **e** Western blot analysis of AGPS protein expression in DU145 and 22Rv1 cells with or without Larotrectinib treatment. **f** Western blot analysis of ubiquitination levels in ectopically expressed Flag-tagged AGPS and ectopically expressed Myc-tagged MDM2 in DU145 cells treated with Larotrectinib. **g** Western blot analysis of ubiquitination levels in ectopically expressed Flag-tagged AGPS and ectopically expressed Myc-tagged MDM2 in DU145 cells treated with MG132 (20 μM) for 8 h or with Larotrectinib. **h** Western blot analysis of AGPS protein expression in DU145 cells with or without CHX (50 μg/mL) treatment for 0-, 4-, 8-, 12-, 16-, or 20- h. **i** Protein bands were quantified and normalized to the band intensity at the 0-h time point. * *P* < 0.05, ** *P* < 0.01, *** *P* < 0.001. **j** Co-IP analysis of binding of Flag-tagged AGPS with ectopically expressed Myc-tagged MDM2 in DU145 cells after NTRK1 knockdown. **k** Western blot analysis of ubiquitination levels in ectopically expressed Flag-tagged AGPS with ectopically expressed Myc-tagged MDM2 in DU145 cells after NTRK1 knockdown and treated with MG132 (20 μM) for 8 h

To future illustrate the regulation mechanism, we screened several common kinases from both databases that might modify the phosphorylation of AGPS at the Y451 position (The sequence of AGPS used was obtained from two separate databases: https://services.healthtech.dtu.dk/service.php?NetPhos-3.1 and www.phosphonet.ca), including EGFR/ERBB4, Ack/TNK2, Met/MSTIR, PDGFR/CFFIP, and Trk/NTRK1. Only the TrkA inhibitor, Larotrectinib, but not any other inhibitor could significantly increase the expression of endogenous AGPS (Fig. 6a). Interestingly, we conducted an intersection analysis between these kinases and AGPS-related proteins in the Biogrid database, revealing that solely TrkA exhibited a connection with AGPS (Supplementary Fig. 4b). This finding further substantiates our observed phenomenon. Furthermore, we discovered that TrkA is overexpressed and phosphorylated in prostate cancer, suggesting a potential association with disease progression. Consequently, we selected the TrkA inhibitor Larotrectinib for further investigation. Also, Co-IP results suggested that Larotrectinib could inhibit the phosphorylation of AGPS tyrosine (Fig. 6b), which was consistent with our previous results that TrkA can modify the phosphorylation of AGPS.

Interestingly, it has been reported that TrkA is aberrantly activated in PCa and the phosphorylation level is positively correlated with the Gleason score [22, 23]. We examined the phosphorylation level of TrkA in PCa cells and also found that p-TrkA was significantly higher in PCa cells than in RWPE-1 cells (Fig. 6c). Immunohistochemistry in prostate tissues also indicated the expression of p-TrkA gradually increased with increasing Gleason score. On the contrary, AGPS expression is a negative correlation with the Gleason score and p-TrkA level (Fig. 6d, Supplementary Fig. 4c). Meanwhile, we found that the expression of AGPS was significantly elevated with p-TrkA inhibitor Larotrectinib (Fig. 6e and f). Also, the ubiquitination level of AGPS was significantly reduced and AGPS protein half-life significantly increased after Larotrectinib treatment which was consistent with our previous findings (Fig. 6g-i). To further corroborate our findings, we knocked down NTRK1 (Supplementary Fig. 4d) and found that the ubiquitination level of AGPS and the binding of AGPS and MDM2 were significantly reduced (Fig. 6j, k). This indicated that TrkA phosphorylation modification of AGPS promoted the binding of AGPS and MDM2, leading to the degradation of AGPS. Likewise, the protein half-life of AGPS was increased (Supplementary Fig. 4e and f).

Overall, TrkA is aberrantly activated in PCa and modifies the phosphorylation of AGPS at Y451, which promotes the binding of AGPS and MDM2 and significantly downregulates AGPS expression in PCa.

### Larotrectinib promotes the sensitivity of prostate cancer cells to ferroptosis by inhibiting the phosphorylation of TrkA

**Fig. 7:**
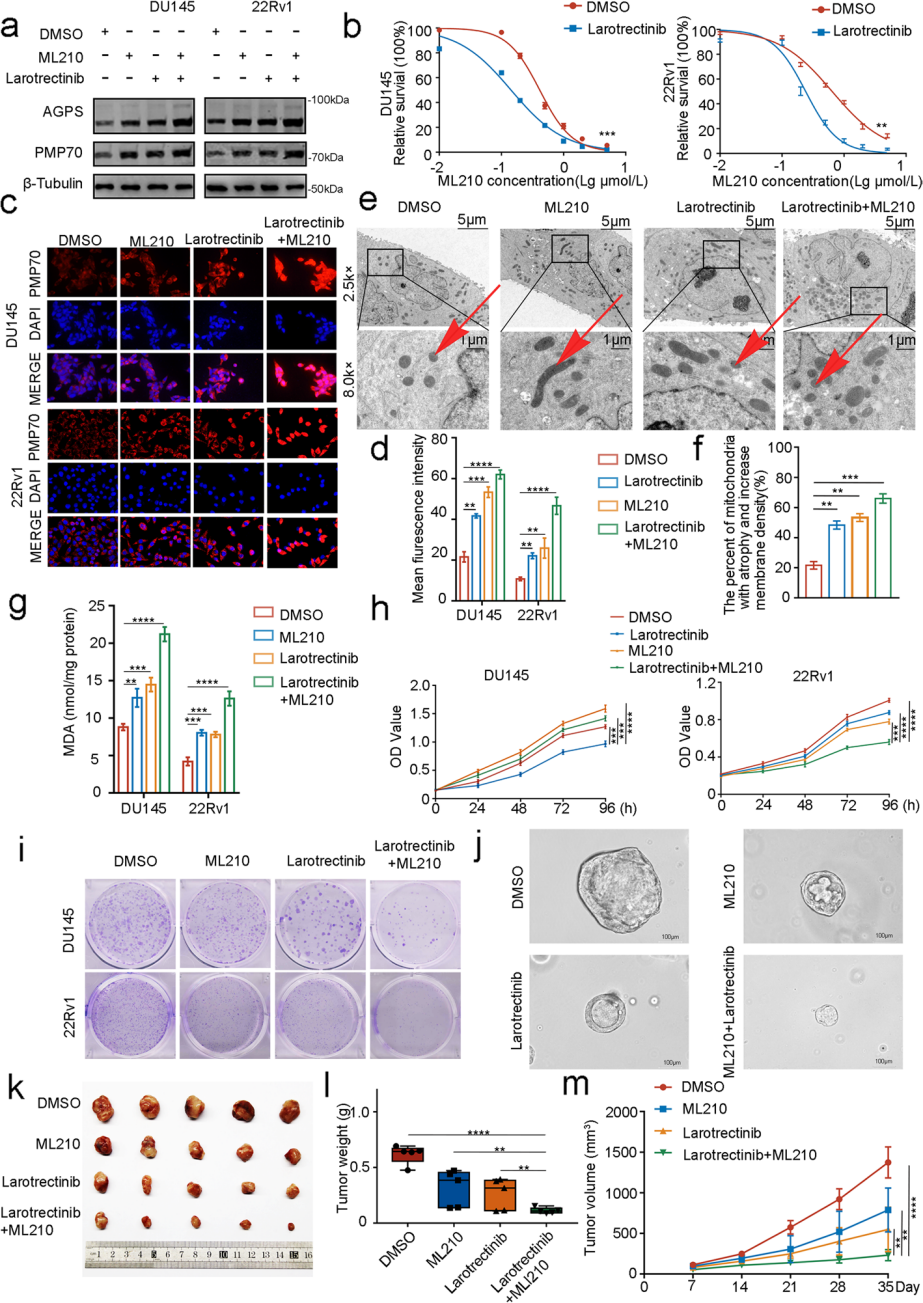
Larotrectinib promotes the sensitivity of prostate cancer cells to ferroptosis by inhibiting the phosphorylation of TrkA. **a** Western blot analysis of AGPS protein expression in DU145 and 22Rv1 cells with or without ML210 (10 μM for 8 h) and Larotrectinib (20 nM, overnight) treatment. **b** The sensitive of ferroptosis in DU145 and 22Rv1 cells with or without ML210 and Larotrectinib treatment. ** *P* < 0.01, *** *P* < 0.001. **c**,** d** PMP70 staining in DU145 and 22Rv1 cells with or without ML210 and Larotrectinib treatment. ** *P* < 0.01, *** *P* < 0.001, **** *P* < 0.0001. **e**,** f** Pca cells were observed by TEM with or without ML210 and Larotrectinib treatment. ** *P* < 0.01, *** *P* < 0.001, **** *P* < 0.0001. **g** MDA level after treatment with ML210 and/or Larotrectinib in DU145 and 22Rv1 cells. * *P* < 0.05, ** *P* < 0.01. **h** CCK8 essay with the OD values in 6-wells plate after treatment with ML210 and/or Larotrectinib in DU145 and 22Rv1 cells. ** *P* < 0.01, *** *P* < 0.001, **** *P* < 0.0001. **i** Colony formation assay displayed the DU145 and 22Rv1 cell colony numbers after treatment with ML210 and/or Larotrectinib. **j** Organoid culture treated with ML210 and/or Larotrectinib from Pca patient tissues. **k** PCa cells were injected subcutaneously into the right flank of mice after treatment with ML210(50 mg/kg) and/or Larotrectinib (300 mg/kg) every other 24 h. Xenograft growth was measured every other day for 28 days. Tumors in each group at day 28 were harvested and photographed. **l** Tumor weight in different groups. Data represented as mean ± SD (*n* = 5). **P < 0.01, **** *P* < 0.0001. **m** Tumor volume at each time point. Data represented as mean ± SD (*n* = 5). n.s., no significance; *P < 0.01, **P < 0.01, ***P < 0.001, **** *P* < 0.0001

In recent years, the application of ferroptosis attracted a lot of attention in tumor therapy, some studies reported the use of ferroptosis inducers RSL3 and Erastin in the treatment of PCa [24]. Therefore, we speculate Larotrectinib could promote the accumulation of AGPS and exert a positive effect on ferroptosis inducers in tumor therapy. To validate our claim, we treated the cells with ML210 and Larotrectinib alone or in combination. We noticed different degrees of elevation of AGPS with the combination application of both drugs (Fig. 7a, Supplementary Fig. 5b). Also, we found that the prostate cancer cells were much more sensitive to ferroptosis inducer when combination administration of Larotrectinib (Fig. 7b, Supplementary Fig. 5c). In addition, PMP70 level and membrane density, and atrophy mitochondria reduced were greatly increased in the combination application of ML210 and Larotrectinib (Fig. 7c-f, Supplementary Fig. 5e). Also, MDA level also increased when combination application of ML210 and Larotrectinib (Fig. 7g, Supplementary Fig. 5f). Thus, we conclude that Larotrectinib-induced accumulation of AGPS could significantly increase the sensitivity of PCa cells to ferroptosis, and this was more pronounced when ML210 was used concomitantly.

Next, we examined the effect of both drugs on cell proliferation. The CCK8 assay and colony formation results suggested that the inhibition of cell proliferation by the combination of both drugs was significantly higher than the individual drug treatments (Fig. 7h and i, Supplementary Fig. 5a, h and i). To further verify the therapeutic efficacy of the two drugs in vivo, we established organoid culture from prostate cancer tissues, when cultured with different drugs, the result showed that the sphere area of organoids generated through the synergistic application of two drugs exhibits a significantly reduced magnitude compared to the individual drug effects, aligning with the outcomes obtained from our in vitro experimental investigations (Fig. 7j, Supplementary Fig. 5j).Finally, we performed a subcutaneous transplantation tumor model using severely immune-deficient mice and found that the tumor suppression effect of Larotrectinib in combination with ML210 was much greater than that of the individual drug treatments (Fig. 7k-m).

In conclusion, Larotrectinib can increase the sensitivity of PCa cells to ferroptosis by promoting the accumulation of AGPS, thereby enhancing the therapeutic effect of ferroptosis inducers on tumors. This provides new ideas for the clinical treatment of PCa.

## Discussion

**Fig. 8:**
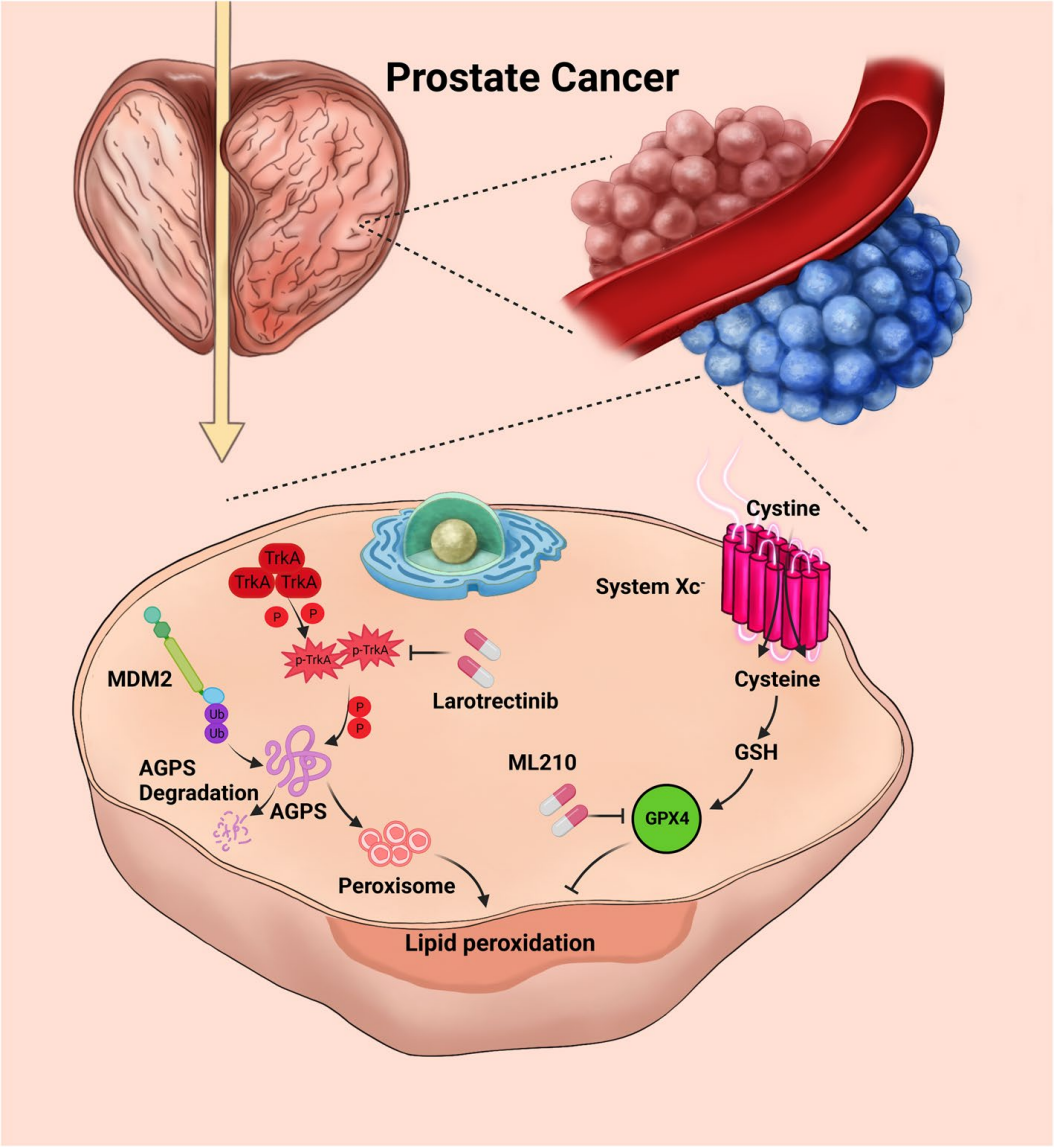
A hypothetic model depicting the dual regulation mechanism of AGPS protein degradation by MDM2 and TrkA inhibitor. MDM2 ubiquitinates and promotes the degradation of AGPS, phosphorylation kinase TrkA aberrantly activated in prostate cancer and facilitates MDM2 ubiquitinate AGPS. TrkA inhibitor Larotrectinib could reactivate peroxisome pathway and sensitive prostate cancer cells to ML210. The combined administration of ML210 and Larotrectinib could significantly ablate the progression of prostate cancer.The model was created by Pro Procreate software and BioRender website (www.biorender.com)

Previous studies focused on the role of AGPS in the synthesis of ether lipids, which affects the progression of various diseases by regulating lipid synthesis. It has been reported that AGPS can inhibit the progression of hepatocellular carcinoma and glioma by regulating the cell cycle and the activity of signal transduction pathways such as MAPK [25]. In contrast, there are very few reports related to the role of AGPS in peroxisome production and tumor progression. In the synthesis of peroxisomes, AGPS promotes peroxisome production via the activation of GNPAT [26]. Our study revealed that AGPS inhibited the proliferation of PCa cells by promoting peroxisome production, thereby leading to lipid peroxidation in PCa cells and enhancing the process of ferroptosis (Fig. 8).

Ferroptosis is iron-dependent cell death caused by lipid peroxidation due to excessive accumulation of ROS [10]. In this process, GPX4 is mainly responsible for degrading lipid peroxides and removing toxic intermediates. The inhibition of GPX4 initiates ferroptosis. The development of small molecule covalent inhibitors of GPX4 to effectively target cancer cells is appealing in modern drug development [18]. The currently available GPX4 inhibitors, such as RSL3 and ML162, exert their inhibitory effects by covalently linking the selenocysteine residue at position 46 on the active site of GPX4 through a reactive alkyl chloride moiety. ML210, a molecule with a nitro isoxazole structure, is widely used for inducing ferroptosis [27]. Researchers have explored the antitumor molecular mechanism of ML210 to inhibit GPX4 and reported a tumor-suppressive effect in a variety of tumors [28]. Our study also verified that ML210 could achieve the therapeutic effect of inhibiting PCa progression by promoting ferroptosis. However, its therapeutic effect is limited because of the complex ferroptosis process, thus new therapeutic modalities need to be further explored (Fig. 8).

TrkA is a tyrosine kinase receptor and is closely related to the progression of many diseases. Its activation can activate pathways such as AKT for disease development [23]. TrkA is highly phosphorylated in a variety of tumors, and its inhibitors are effective in tumor suppression. Among them, Larotrectinib has been clinically used as an inhibitor of TrkA in various tumors such as liver cancer and breast cancer [29]. Our study revealed that in PCa, abnormally activated TrkA promoted the degradation of AGPS via MDM2. Therefore, the inhibition of TrkA could inhibit the degradation of AGPS via MDM2 and enhance the sensitivity of PCa cells to ML210. The combination of the TrkA inhibitors Larotrectinib and ML210 increased the lethality of ML210 and Larotrectinib towards PCa cells (Fig. 8). Our study provided a theoretical basis for the use of Larotrectinib in the treatment of PCa in the clinical setting.

MDM2 often appears as a complex with p53 in vivo and could suppress ferroptosis by degrading p53 [30–33]. Since tumor Suppressor p53 and its mutants have widely reported involved in ferroptosis development and progression [34, 35]. This will undoubtedly lead to the consideration of whether MDM2 affects ferroptosis through p53 and whether AGPS are involved in this process. In order to eliminate the influence of p53 as far as possible, many experiments were carried out in both p53 positive (22Rv1, DU145) and p53 negative cell lines (PC-3), which including the detection of peroxisome level, ferroptosis level and mitochondrial membrane potential. These findings suggested that under the condition of p53 deletion, MDM2-AGPS can still affect the occurrence of ferroptosis by regulating the formation of peroxisome and ferroptosis inducer drug sensitivity in a p53 independent manner. Since ferroptosis involves in different processes and molecular pathways, further research is needed to better understand the mechanism of MDM2 and AGPS in ferroptosis.

## Conclusion

Over all these findings, our study identified AGPS as a novel substrate of MDM2. Their interaction could be regulated by TrkA and its inhibitor, Larotrectinib. Larotrectinib could enhance the sensitivity of PCa cells to ML210, and the combined application of Larotrectinib and ML210 could achieve a better anti-tumor effect on PCa. Our study is important for exploring new clinical treatment modalities for PCa.

### Supplementary Information

**Additional file 1:** **Supplementary Fig 1.** (**Supplemental to Fig. 1**) AGPS is down-regulated in prostate cancer and negatively correlated with prognosis. **Supplementary Fig 2.** (**Supplemental to Fig. 2**) AGPS inhibits the proliferation of PC-3 cells. **Supplementary Fig 3.** (**Supplemental to Fig. 3**) MDM2 inhibits ferroptosis through regulating AGPS- a p53-independent pathway. **Supplementary Fig 4.** (**Supplemental to Fig. 6**) TrkA has a significant effect on the stability of the protein of AGPS. ** Supplementary Fig 5.** (**Supplemental to Fig. 7**) Effectiveness of the combination of  ML210 and Larotrectinib, in the PC3 cell line. **Supplementary Table 1.**Oligonucleotides used for relative gene expression by qRT-PCR. **Supplementary Table 2.** The oligonucleotides of si- or sh- RNAs.

## Data Availability

The raw data generated in this study are available upon request, from the corresponding author.

## Abbreviation

PCa: Prostate cancer
AGPS: Alkylglycerone phosphate synthase Gene
MDM2: Mousedouble minute 2
TrkA: Tyrosine kinase receptor A
IHC: Immunohistochemistry
RT-qPCR: Real-time quantitative polymerase chain reaction
TEM: Transmission electron microscope
