# Supplementary material for: TrkA promotes MDM2-mediated AGPS ubiquitination and degradation to trigger prostate cancer progression
Source: J Exp Clin Cancer Res. 2024 Jan 11;43:16. doi: 10.1186/s13046-023-02920-w (PMC10782585; doi:10.1186/s13046-023-02920-w)
Supplement: Supplementary file 1 — Additional file 1: Supplementary Fig 1. (Supplemental to Fig. 1) AGPS is down-regulated in prostate cancer and negatively correlated with prognosis. Supplementary Fig 2. (Supplemental to Fig. 2) AGPS inhibits the proliferation of PC-3 cells. Supplementary Fig 3. (Supplemental to Fig. 3) MDM2 inhibits ferroptosis through regulating AGPS- a p53-independent pathway. Supplementary Fig 4. (Supplemental to Fig. 6) TrkA has a significant effect on the stability of the protein of AGPS. Supplementary Fig 5. (Supplemental to Fig. 7) Effectiveness of the combination of ML210 and Larotrectinib, in the PC3 cell line. Supplementary Table 1.Oligonucleotides used for relative gene expression by qRT-PCR. Supplementary Table 2. The oligonucleotides of si- or sh- RNAs. [file 13046_2023_2920_MOESM1_ESM.docx]

**TrkA promotes MDM2-mediated AGPS ubiquitination and degradation to trigger prostate cancer progression**

*Yu Zhang, Zhenlin Huang, Keqiang Li, Guoqing Xie, Yuankang Feng,Zihao Wang, Ningyang Li, Ruoyang Liu,Yinghui Ding, Jun Wang, Jinjian Yang , Zhankui Jia*

**Files included in the supplementary information**

**Supplementary Fig 1 （Supplemental to Fig. 1）**AGPS is down-regulated in prostate cancer and negatively correlated with prognosis.

**Supplementary Fig 2 （Supplemental to Fig.2）**AGPS inhibits the proliferation of PC-3 cells.

**Supplementary Fig 3 （Supplemental to Fig. 3）**MDM2 inhibits ferroptosis through regulating AGPS- a p53-independent pathway.

**Supplementary Fig 4 （Supplemental to Fig. 6）**TrkA has a significant effect on the stability of the protein of AGPS.

**Supplementary Fig 5 （Supplemental to Fig. 7）**Effectiveness of the combination of ML210 and Larotrectinib, in the PC3 cell line.

**Supplementary Table1.** Oligonucleotides used for relative gene expression by qRT-PCR.

**Supplementary Table 2.** The oligonucleotides of si- or sh- RNAs.


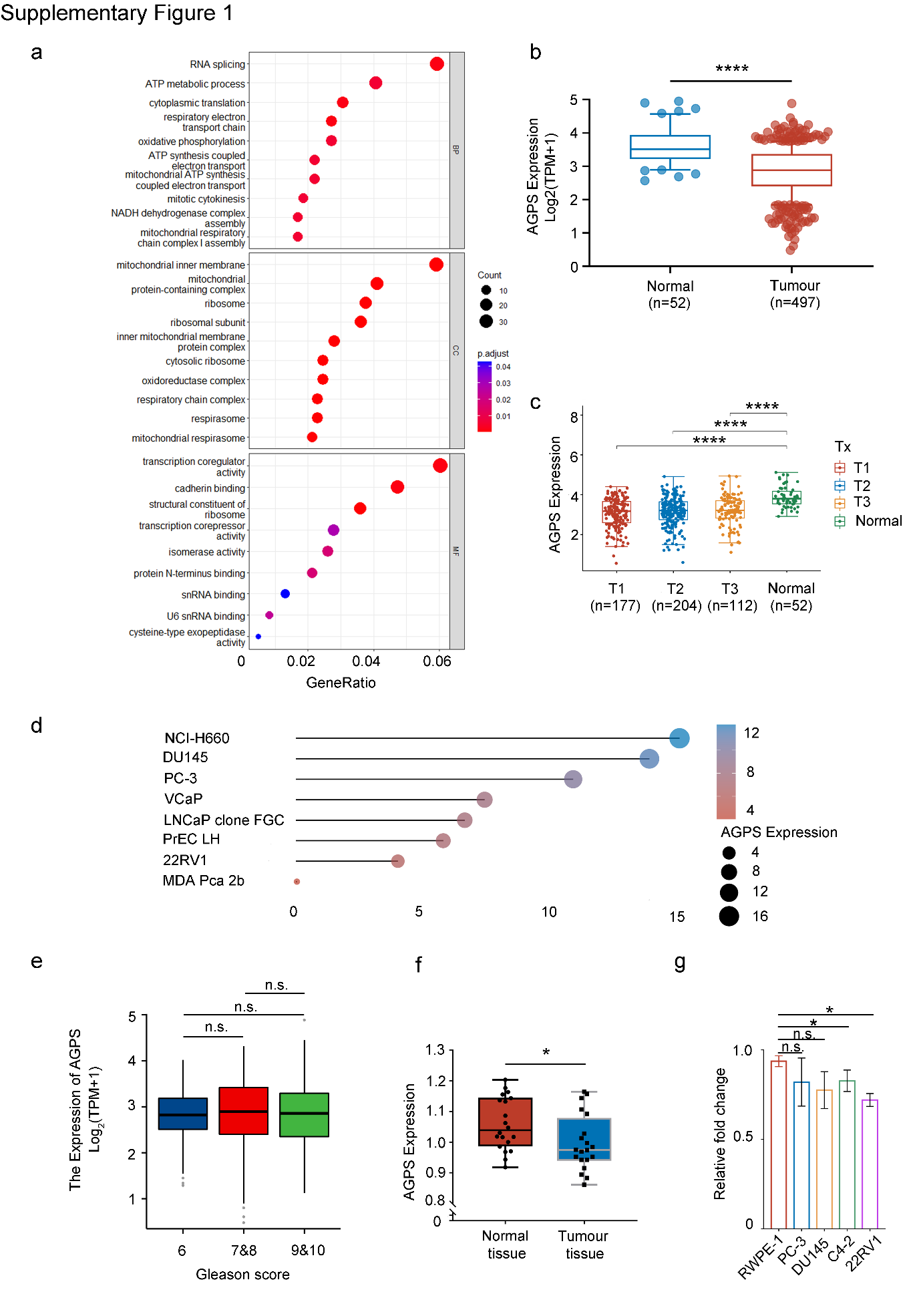


**Supplementary Fig 1.**

**a**. The GO enrichment analysis of the Label-Free Mass spectrometry data. **b**. AGPS expression in mRNA level from TCGA database between PCa tumor tissues and normal tissues. **** *P* < 0.0001 **c**. AGPS expression on mRNA level on TCGA database in different PCa T stages. **** *P* < 0.0001. **d**. AGPS expression on mRNA level from CCLE (<https://sites.broadinstitute.org/ccle/>) database in different PCa cell lines. **e**. AGPS mRNA expression with different Gleason scores. n.s. no significance. **f**. AGPS mRNA expression in PCa tissues and normal tissues. * *P* < 0.05. **g**. AGPS mRNA expression in different PCa cell lines. n.s. no significance. * *P* < 0.05.

**
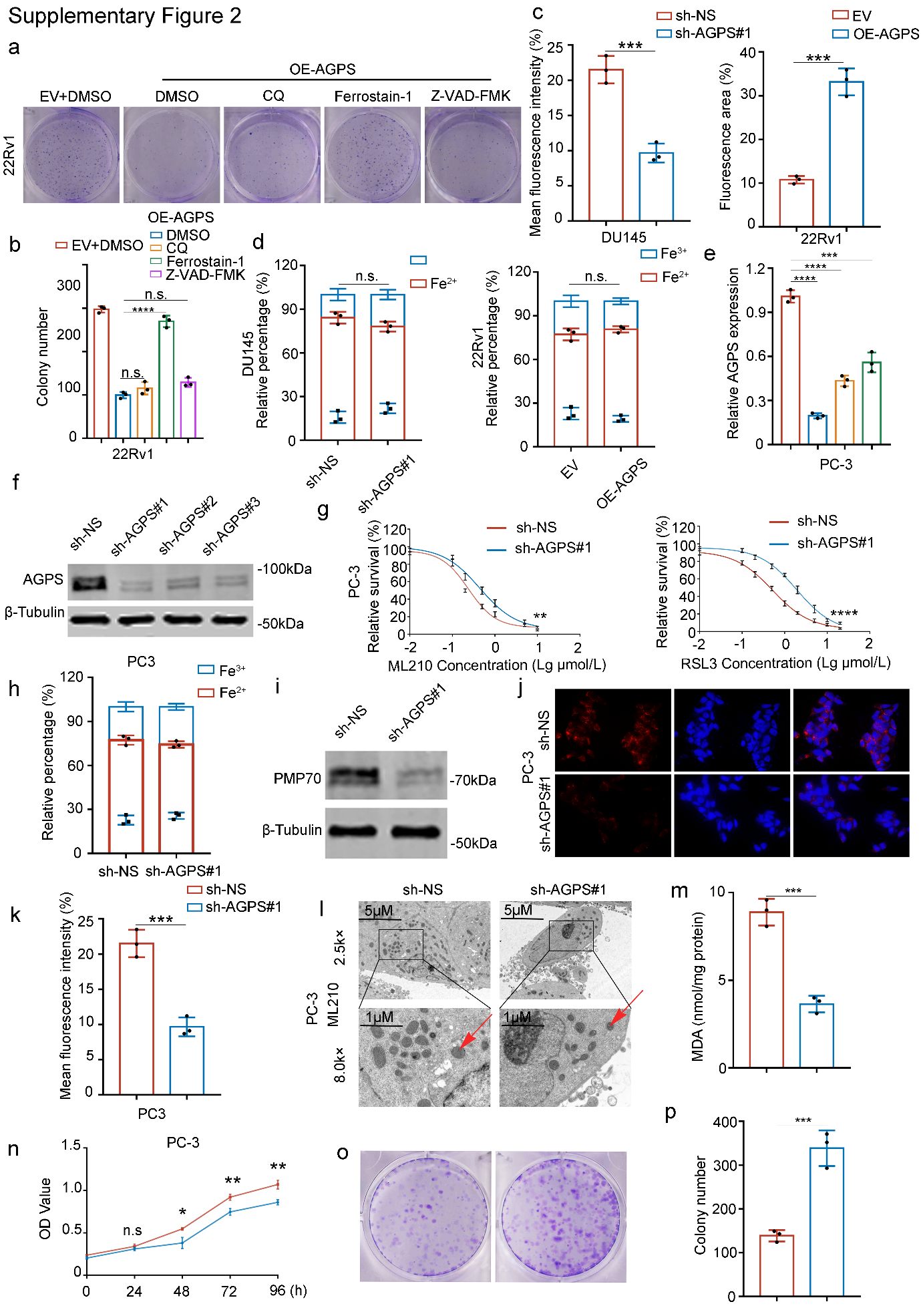
**

**Supplementary Fig 2.**

**a,b**. The colony formation experiment demonstrated cell proliferation subsequent to the overexpression of AGPS in the 22Rv1 cell line, coupled with the administration of autophagy inhibitor (CQ,25μM), ferroptosis inhibitor (Ferrostain-1,1μM), and apoptosis inhibitor (Z-VAD-FMK,40μM), individually from the 3rd day. n.s. no significance. **** *P* < 0.0001. **c**. Mean fluorescence intensity of PMP70 after AGPS knockdown in DU145 cells and overexpression in 22Rv1 cells. *** *P* < 0.001. **d**. Changes of Fe^2+^ and Fe^3+^ in DU145 cells after AGPS knockdown and in 22Rv1 cells after AGPS overexpression. n.s. no significance. **e**. Knocking down efficiency of AGPS in PC-3 cells detected by RT-qPCR. *** *P* < 0.001, **** *P* < 0.0001. **f**. Knocking down efficiency of AGPS in PC-3 cells detected by western blot. **g**. IC50 values of ML210 and RSL3 in PC-3 cells after AGPS knockdown. ** *P* < 0.01, **** *P* < 0.0001. **h**. Changes of Fe^2+^ and Fe^3+^ in PC3 cells after AGPS knockdown. n.s. no significance. **i**. MP70 protein expression after AGPS knockdown with shRNA in PC-3 cells. **j**. PMP70 protein immunofluorescence staining after AGPS knockdown with shRNA in PC-3 cells. **k**. Mean fluorescence intensity of PMP70 after AGPS knockdown in PC-3 cells. *** *P* < 0.001. **l**. PCa cells were observed by TEM when induced with ML210 after AGPS knockdown. **m.** MDA levels after AGPS knockdown with shRNA in PC-3 cells. **n**. CCK8 assay on the OD value in 6-well plates after AGPS knockdown with shRNA in PC-3cells. **o, p**. Colony formation assay on colony numbers of the PC-3 cells after AGPS knockdown with shRNA.

**
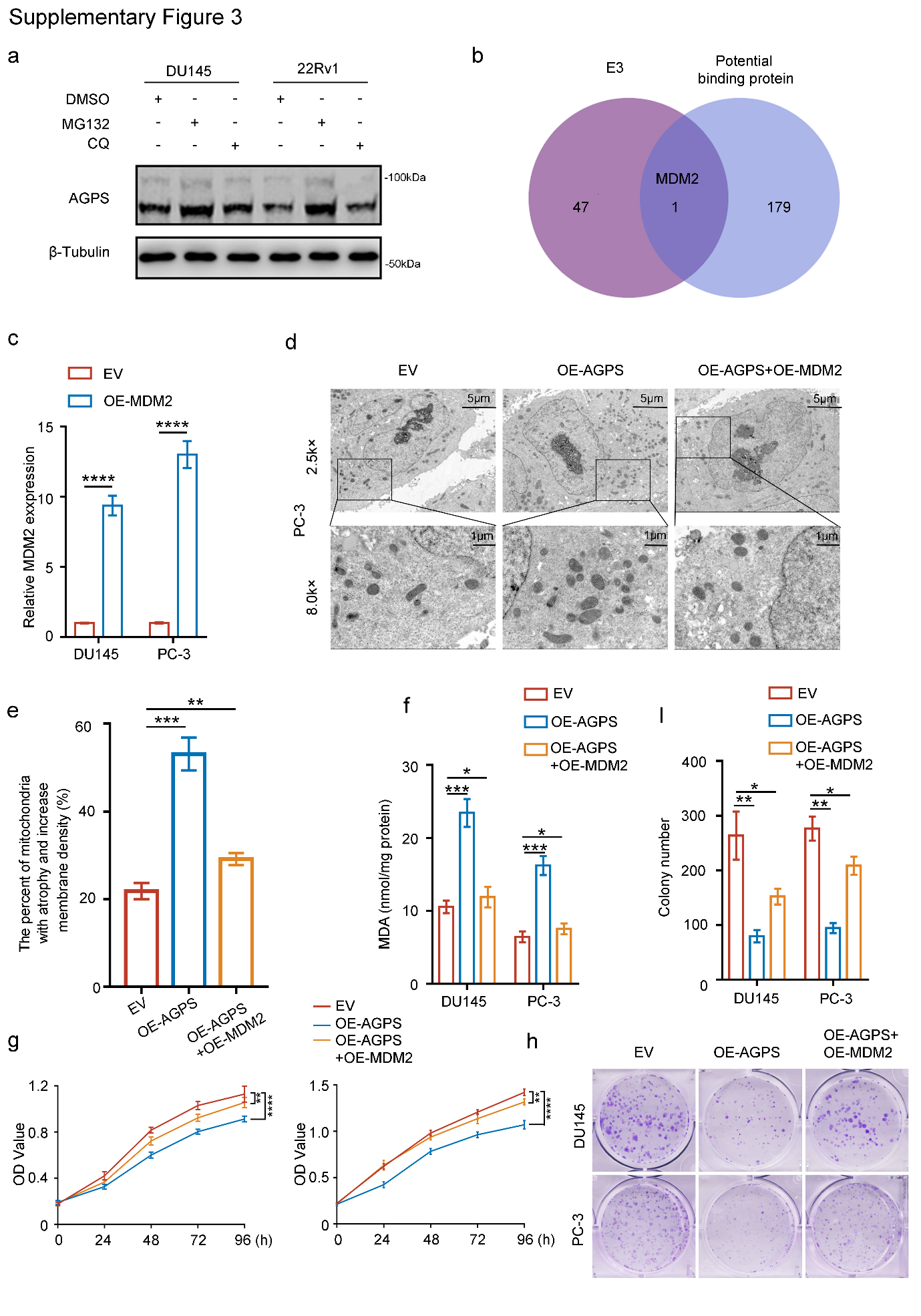
**

**Supplementary Fig 3.**

**a.** Western blot of AGPS protein expression after being treated with proteasome inhibitor MG132 (20mM, 8 hours) and lysosome inhibitor chloroquine (CQ, 25μM, overnight). **b**. Venn diagram shows the intersection of the possible E3 enzymes predicted from the theUbibrowser2 database for AGPS and the proteins predicted from the Biogrid database that may interact with AGPS. **c**. MDM2 mRNA expression after overexpression of MDM2. **** *P* < 0.0001. **d, e**. PCa cells were observed by TEM. **f**. MDA level after AGPS overexpression and MDM2 overexpression. * *P* < 0.005, *** *P* < 0.001.**g.** CCK8 assay measures the OD value in 6-well plates after AGPS overexpression and MDM2 overexpression. **h, i**. Colony formation assay on colony numbers of the DU145 and PC-3 cell after AGPS overexpression and MDM2 overexpression. * *P* < 0.05. ** *P* < 0.01.

**
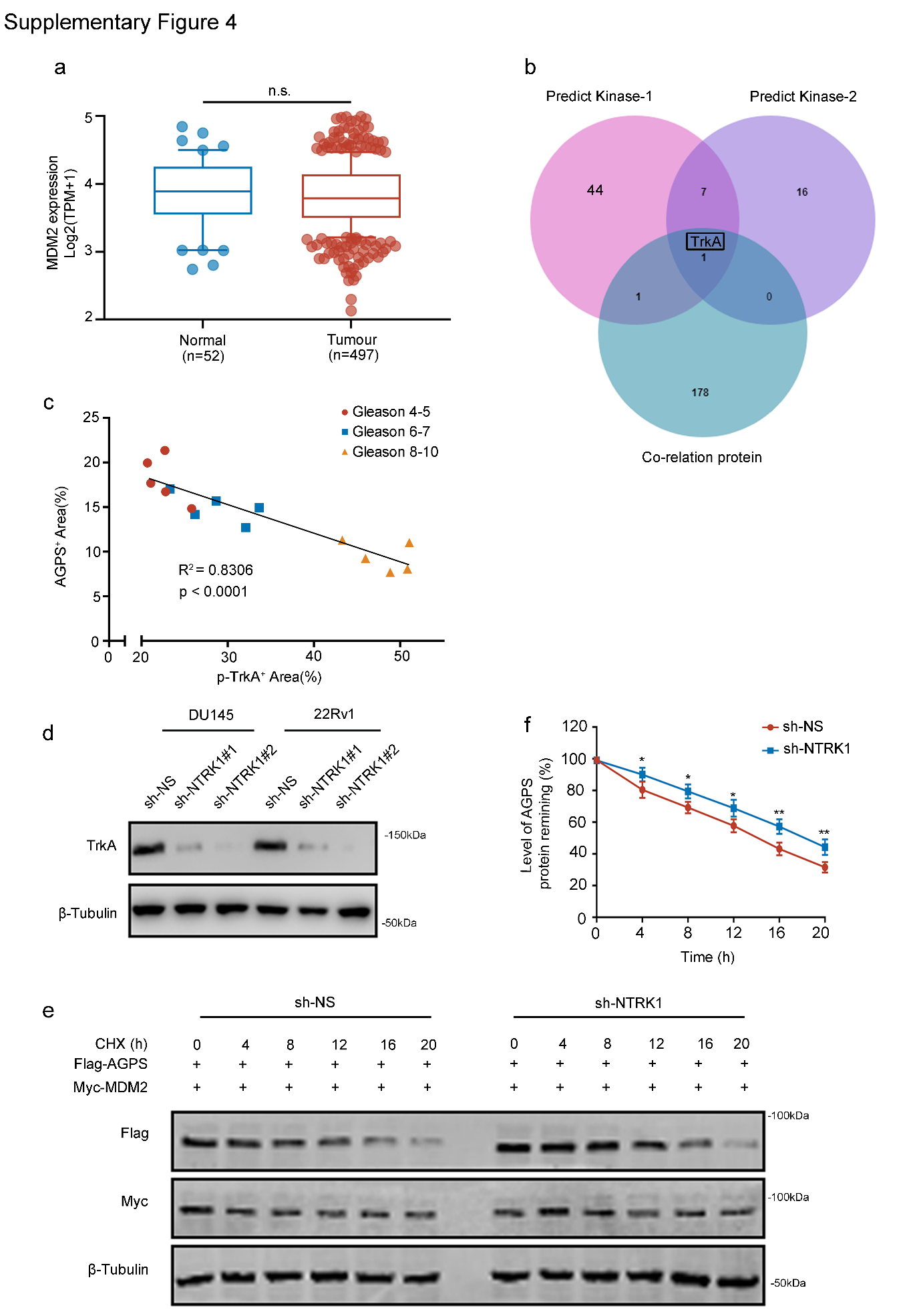
**

**Supplementary Fig 4.**

**a.** MDM2 mRNA expression from TCGA database. **b.** Venn diagram shows the intersection of predicted kinases and interacting proteins. **c**. Co-relationship between AGPS and p-TrkA. **d**. Knock-down efficiency of TrkA. **E, f**. Changes in protein levels after TrkA knocked-down with CHX at 4-, 8-, 12-, 16-, and 20- hours, respectively. ** *P* < 0.01.


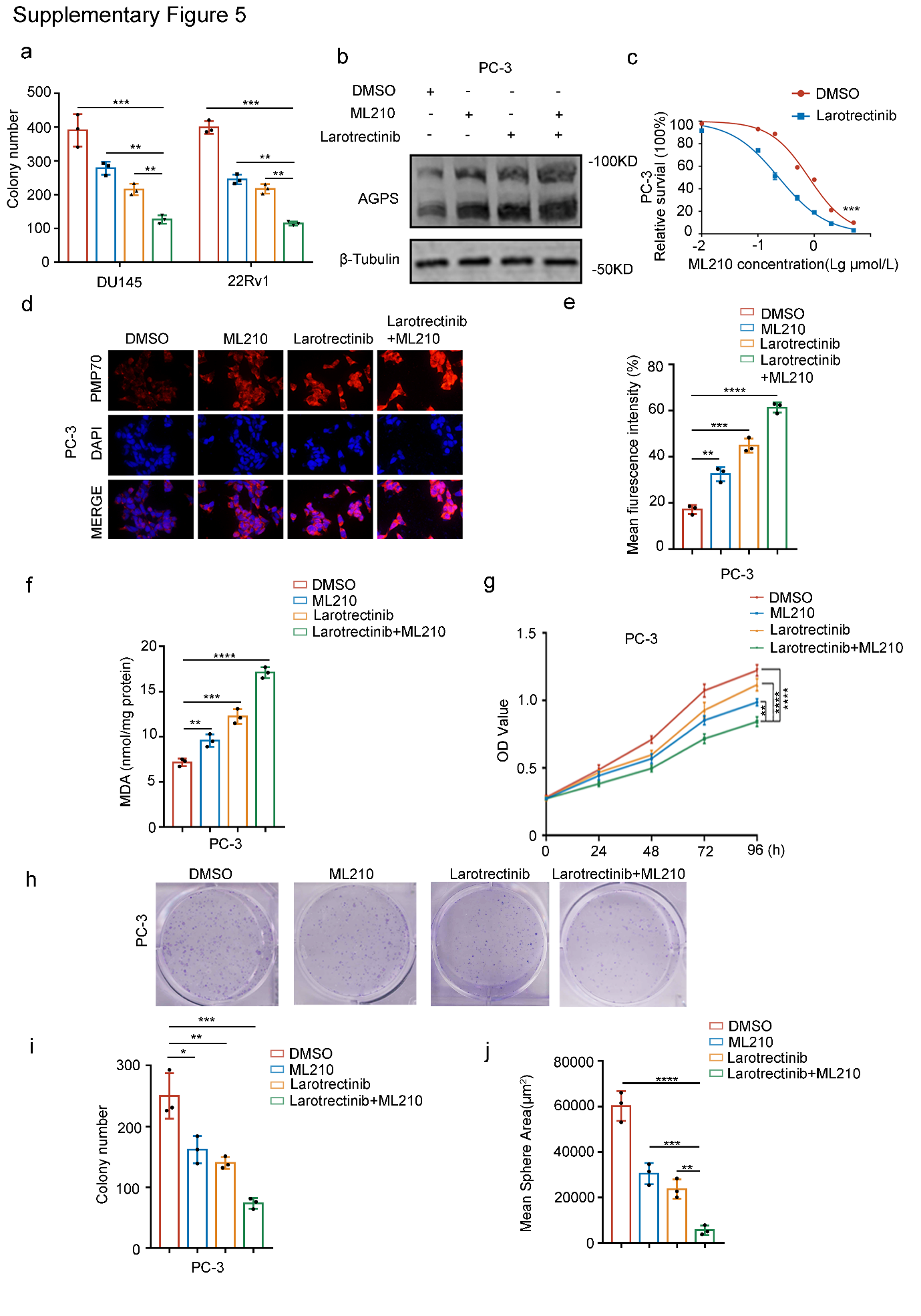


**Supplementary Fig 5.**

**a.** Statistic of colony formation assay after treatment with ML210 and/or Larotrectinib in PC-3 cells. ** *P* < 0.01, *** *P* < 0.001. **b.** Western blot analysis of AGPS protein expression in PC-3 cells with or without ML210 and Larotrectinib treatment. **c**. The sensitive of ferroptosis in PC-3 cells with or without ML210 and Larotrectinib treatment. *** *P* < 0.001. **d, e**. PMP70 staining in PC-3 cells with or without ML210 and Larotrectinib treatment. ** *P* < 0.01, *** *P* < 0.001, **** *P* < 0.0001. **f**. MDA level after treatment with ML210 and/or Larotrectinib in PC-3 cells. ** *P* < 0.01, *** *P* < 0.001, **** *P* < 0.0001. g. CCK8 essay with the OD values in 6-wells plate after treatment with ML210 and/or Larotrectinib in PC-3 cells. ** *P* < 0.01, **** *P* < 0.0001. h, i. Colony formation assay displayed the PC-3 cell colony numbers after treatment with ML210 and/or Larotrectinib. * *P* < 0.05, ** *P* < 0.01, *** *P* < 0.001. j. Statistic of organoid culture treated with ML210 and/or Larotrectinib from Pca patient tissues. ** *P* < 0.01, *** *P* < 0.001, **** *P* < 0.0001.

**Supplementary Table 1. Oligonucleotides used for relative gene expression by qRT-PCR.**

| Target gene | Forward primers (5’→3’) | Reverse primers (5’ →3’) |
| --- | --- | --- |
| AGPS | TAGGAGGATGGGTATCTACTCG | GATATCAGGGCCTGTTGACATA |
| MDM2 | AGGCAGGGGAGAGTGATAC  AGATTC | CAGGAAGCCAATTCTCACG  AAGGG |
| ACTB | CCTGGCACCCAGCACAAT | GGGCCGGACTCGTCATAC |

**Supplementary Table 2.** **The oligonucleotides of siRNA or shRNA.**

| Product name | Forward primers (5’-3’) |
| --- | --- |
| AGPS shRNA-1 | GTGACCCACTGACCGTATTTG |
| AGPS shRNA-2 | GGCATGGGTTTACCAACATTT |
| AGPS shRNA-3 | TTCAGAATAACGAATATT |
| MDM2 siRNA-1  MDM2 siRNA-2  MDM2 siRNA-3  NTRK1 shRNA-1  NTRK1 shRNA-2 | AAGCCAUUGCUUUUGAAGUUA* AAGATACCAGATCATGTCAGA*  CTCAGCCATCAACTTCTAGTA*  TATCTACAGCACCGACTATTA  AGTCAGCCACGGTGATGAAAT; |

* The oligonucleotides from WeizhenBio, Shandong, China.
